# IL-21 and anti-**α**4**β**7 dual therapy during ART promotes immunological and microbiome responses in SIV-infected macaques

**DOI:** 10.1172/jci.insight.184491

**Published:** 2025-02-04

**Authors:** Samuel D. Johnson, Maria Pino, Arpan Acharya, Julien A. Clain, Deepanwita Bose, Kevin Nguyen, Justin Harper, Francois Villinger, Mirko Paiardini, Siddappa N. Byrareddy

**Affiliations:** 1 Department of Pharmacology and Experimental Neuroscience, University of Nebraska Medical Center (UNMC), Omaha, Nebraska, USA.; 2 Division of Microbiology and Immunology, Emory National Primate Research Center (ENPRC), Emory University, Atlanta, Georgia, USA.; 3 New Iberia Research Center, University of Louisiana at Lafayette, New Iberia, Louisiana, USA.; 4 Department of Pathology and Laboratory Medicine, Emory University School of Medicine, Atlanta, Georgia, USA.; 5 Department of Genetics, Cell Biology and Anatomy, and; 6 Department of Biochemistry and Molecular Biology, UNMC, Omaha, Nebraska, USA.

**Keywords:** AIDS/HIV, Immunology, Immunotherapy, Integrins, Microbiome

## Abstract

Despite combination antiretroviral therapy (ART), HIV causes persistent gut barrier dysfunction, immune depletion, and dysbiosis. Furthermore, ART interruption results in reservoir reactivation and rebound viremia. Both IL-21 and anti-α4β7 improve gut barrier functions, and we hypothesized that combining them would synergize as a dual therapy to improve immunological outcomes in SIV-infected rhesus macaques (RMs). We found no significant differences in CD4^+^ T cell reservoir size by intact proviral DNA assay. SIV rebounded in both dual-treated and control RMs following analytical therapy interruption (ATI), with time to rebound and initial rebound viremia comparable between groups; however, dual-treated RMs showed slightly better control of viral replication at the latest time points after ATI. Additionally, following ATI, dual-treated RMs showed immunological benefits, including T cell preservation and lower PD-1^+^ central memory T cell (TCM) frequency. Notably, PD-1^+^ TCMs were associated with reservoir size, which predicted viral loads (VLs) after ATI. Finally, 16S rRNA-Seq revealed better recovery from dysbiosis in treated animals, and the butyrate-producing Firmicute *Roseburia* predicted PD-1–expressing TCMs and VLs after ATI. PD-1^+^ TCMs and gut dysbiosis represent mechanisms of HIV persistence and pathogenesis, respectively. Therefore, combining IL-21 and anti-α4β7 may be an effective therapeutic strategy to improve immunological outcomes for people with HIV.

## Abstract 2

HIV causes damage to the gut and immune dysregulation. Targeting these with combined interleukin-21 and anti-α4β7 improved viral, immunological, and microbiome measures in SIV-infected macaques.

## Graphical abstract

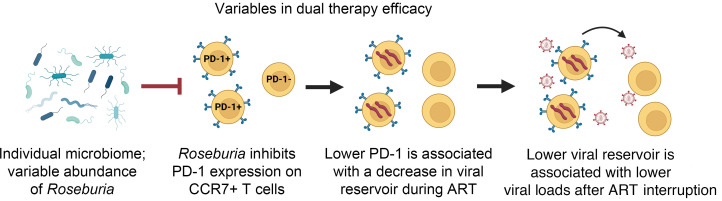

## Introduction

Currently, approximately 40 million people are living with the HIV (PLWH) globally (1). The introduction of combination antiretroviral therapies (ART) has dramatically improved their prognosis, with most individuals who maintain daily treatment experiencing full suppression of viral replication and significant immune reconstitution (2). Despite these improvements, chronic immune activation, mainly due to persistent microbial translocation across the gut barrier, contributes to significant pathology associated with accelerated biological aging, including increased incidence of cardiovascular disease, metabolic dysregulation, and HIV-associated neurocognitive disorders (HAND) (3). Under physiologic conditions, microbial translocation is controlled by gut physical and immunological barriers. Th17 CD4^+^ T cells are essential for maintaining gut integrity and controlling microbial translocation; however, these cells are preferentially depleted during progressive HIV or SIV infection in nonnatural hosts, including rhesus macaques (RMs) and pig-tailed macaques (PMs) (4–8).

In contrast, natural hosts for SIV, such as sooty mangabeys (SMs), limit epithelium damage, Th17 depletion, and resultant microbial translocation (8, 9). Even with ART suppression, Th17 cells and their ability to produce IL-21, are not fully reconstituted during HIV/SIV infection in nonnatural hosts (4–8). One study has shown that exogenous recombinant IL-21 administration improves the maintenance of these Th17 cells (10). Furthermore, pretreatment with IL-21 improves CD8^+^ T cell effector function during HIV infection and potently inhibits viral replication ex vivo (11). However, consistent with chronic viremia in nonprogressive SIV infection in which IL-21–producing Th17 cells are maintained, IL-21 alone does not induce virologic control in the absence of ART, suggesting that supplementary immunotherapy may be necessary for improving gut immune function and controlling viral rebound (4).

Trafficking of immune cells from the periphery to the gut is regulated primarily by their expression of α4β7 integrin, which binds to mucosal vascular addressin cell adhesion molecule 1 (MAdCAM-1), an addressin expressed on the endothelium of the gut mucosa and gut-associated lymphoid tissues (12). MAdCAM-1 is upregulated in inflammatory bowel diseases (IBDs), and blocking the α4β7/MAdCAM-1 interaction with vedolizumab, a humanized monoclonal antibody to α4β7, is an effective therapeutic for IBDs (13). Early studies using nonhuman primates (NHPs) indicated that differences in α4β7 expression on circulating lymphocytes might be responsible for the difference in immune activation levels, particularly in the gut, between the pathogenic progression of SIV infection in Asian-origin macaques (RMs, PMs) compared with the nonpathogenic SIV infection in African-origin natural hosts (SMs) (14). This knowledge was leveraged in later studies demonstrating that blocking α4β7 with a primatized monoclonal antibody (anti-α4β7) was associated with decreases in SIV mucosal transmission and SIV replication in vivo (15, 16). Epidemiologic studies have corroborated this link demonstrating that individuals with lower α4β7 surface expression on CD4^+^ T cells are less susceptible to HIV vaginal transmission (17). However, additional studies using similar SIV-infection models in RMs did not confirm the ability of α4β7 blockade to induce virologic control after ART interruption, suggesting that the efficacy of anti-α4β7 therapy, including its effect on the gut immunologic status, may be virus specific or host specific (18–22). Recent computational viral kinetic modeling studies further suggested that multiple overlapping mechanisms may act independently or synergistically in individual animals administered anti-α4β7 to facilitate virologic control (23).

Previously, we reported a pilot study documenting the safety and immunological response of combination therapy with IL-21 and anti-α4β7, showing no toxicity and no drug-to-drug interactions that limit the bioavailability of either drug in SIV-uninfected RMs (24). We also noted a decrease in the homing of β7^+^ CD4^+^ memory T cells and a reduction in T cell proliferation in treated animals’ periphery and gut tissues (24). With these encouraging findings, we tested whether IL-21 + anti-α4β7 dual therapy would synergize in reducing inflammation and viral replication in the gut, lowering peripheral viral load (VL), and improving immune functionality. We also performed 16S rRNA-Seq to characterize microbiome compositional changes during the experiment. Herein, we show increased peripheral CD4^+^ and CD8^+^ T cell counts, reduced expression of PD-1 on TCM cells, and a slightly improved viral control in dual-treated, SIV-infected RMs compared with controls. These reductions in VL were closely associated with the size of the peripheral viral reservoir prior to dual therapy. Additionally, the expression of PD-1 and the extent of viral control during ATI correlates with the relative abundance of butyrate-producing *Roseburi*a in the fecal microbiome. These findings support the role of IL-21 and anti-α4β7 dual therapy in promoting immunological and microbiome responses in SIV-infected macaques and provide rationale for additional adjunctive therapies.

## Results

### Anti-α4β7 and IL-21–Fc dual-treated RMs showed a moderately improved control of viral replication at the latest time points following ART interruption.

**Figure 1:**
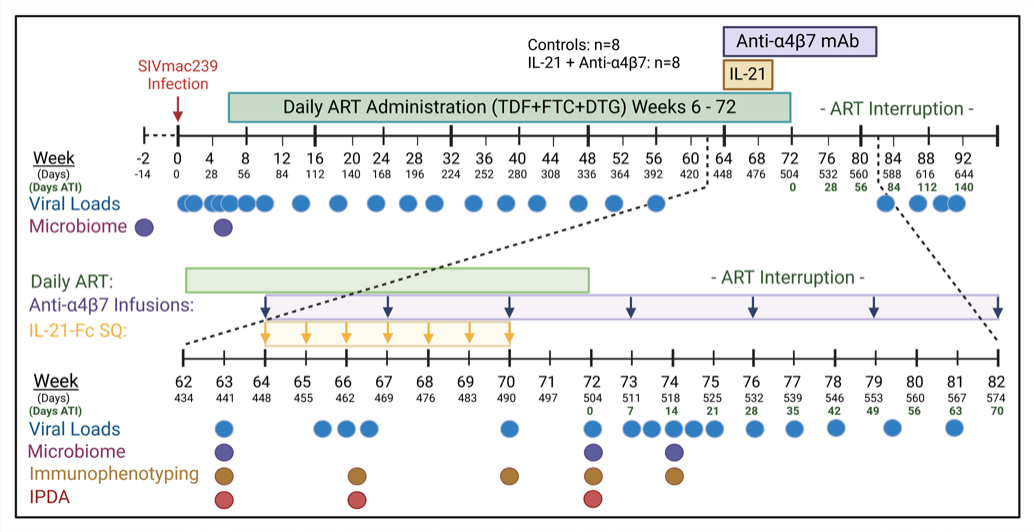
Experimental schema. Sixteen RMs were infected with 300 TCID_50_ SIVmac239 (day 0). On day 42 (week 6) p.i., daily ART (tenofovir disoproxil fumarate, emtricitabine, and dolutegravir) administration was initiated and continued until day 504. On day 448 p.i., dual therapy began for 8 RMs, which included weekly IL-21–Fc administered s.c. for 7 administrations (day 490 p.i.) and anti-α4β7 mAb infusions performed every 3 weeks until day 574 (day 70 ATI; 7 administrations). At day 504 p.i., analytic ART interruption began (day 0 ATI). Longitudinal plasma viral loads were determined from blood collected on days listed.

Sixteen RMs were infected intravenously with 300 TCID_50_ SIV clone SIVmac239 and initiated on ART at day 42 postinfection (p.i.). RMs were subsequently divided into 2 groups of 8 RMs each. One group served as control, and the treated group received s.c. IL-21–Fc weekly (every 7 days) and received anti-α4β7 mAb infusions once every 3 weeks (every 21 days) for a total of 7 administrations of each therapy beginning at day 448 (week 64) p.i. ART was discontinued in all animals for analytic treatment interruption (ATI) at day 504 p.i. (Figure 1).

**Figure 2:**
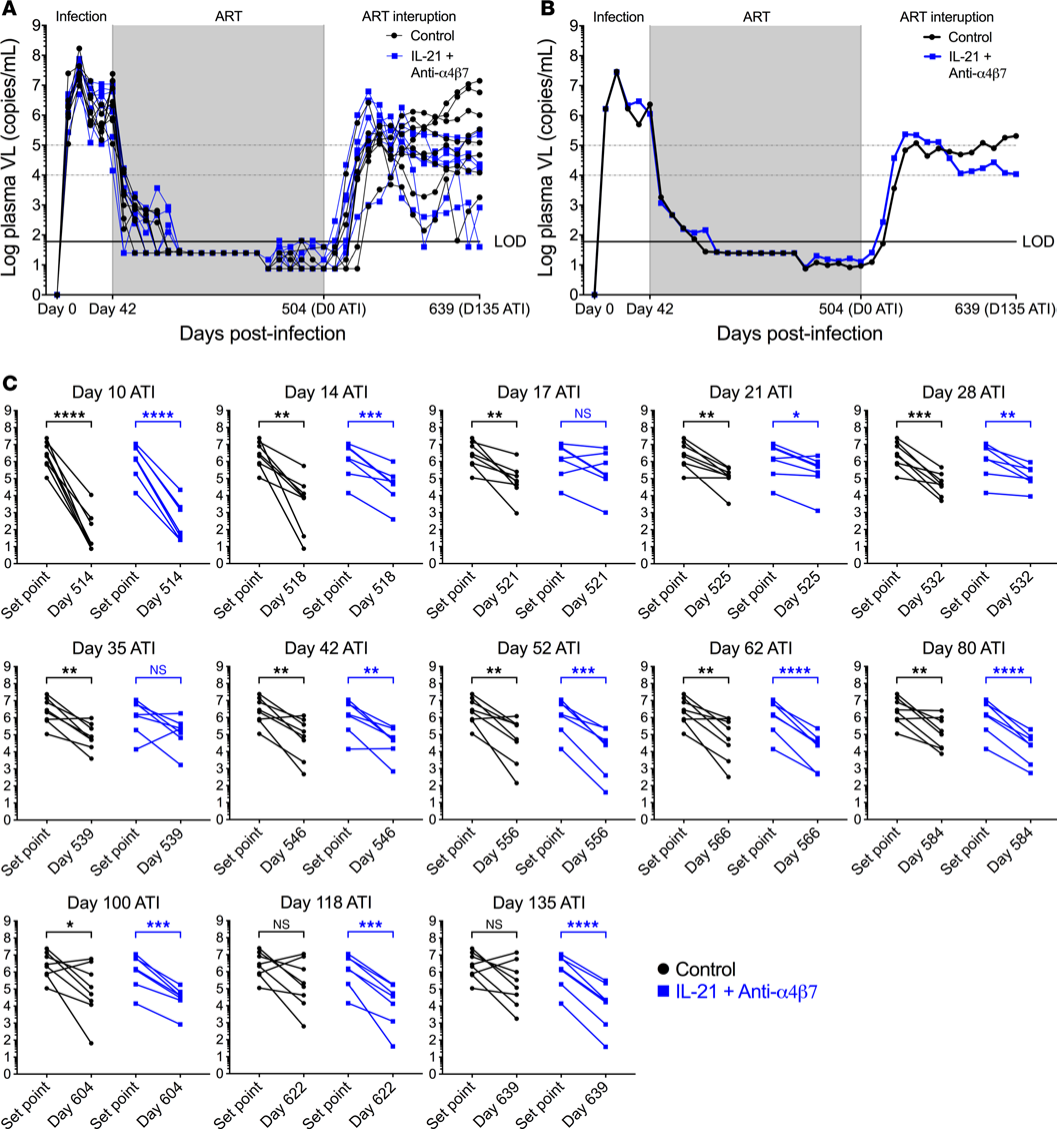
Dual therapy affects viral control. (**A**) Plasma viral loads were measured by qPCR longitudinally for all RMs and reported in copies/mL. Beginning at day 441, a more sensitive hybrid quantitative/digital PCR was utilized. (**B**) Geometric means were calculated according to experimental grouping. (**C**) Ratio paired *t* tests compared viral set point and ATI viral loads. By day 135 ATI (day 639 p.i.), the viral load geometric mean was 3.4 log_10_ lower in the dual-treated group compared with its set point prior to therapy. Data were analyzed by ratio paired *t* tests. *P < 0.05, **P < 0.01, ***P < 0.001, ****P < 0.0001.

Animals were stratified to ensure equivalent peak VLs, set point VL at ART initiation, and time to suppression on ART, with all animals reaching levels of undetectable viremia by day 190 p.i., 148 days after ART initiation (Figure 2A). ATI was initiated at day 504 p.i., until all macaques (except macaque 14_13; discussed below) maintained suppression. Following ATI, both groups experienced viral rebound (Figure 2B). The control group maintained a significantly lower set point VL (geometric mean) compared with before ART until day 100 after ATI, after which point values were no longer significantly different as the mean VL continued to increase. In contrast, dual-treated RMs lost significance from setpoint more rapidly but regained it by day 42 after ATI. At this point, VL levels remained significantly lower than pre-ART values until day 135, the latest assessed time point (Figure 2C). The difference in plasma VL between the control and treated groups was not significant despite opposing trends in VL following peak rebound after ATI. Notably, at the last measurement, the difference in geometric mean between control and experimental animals was log_10_ 1.4 copies/mL, with the dual-treated group showing approximately log_10_ 3.3 copies/mL reduction from peak VL prior to ART, as compared with log_10_ 1.9 in controls. One macaque (14_13) in the treated group was excluded from statistical analysis between groups because it experienced ART failure prior to ATI (Supplemental Figure 1; supplemental material available online with this article; https://doi.org/10.1172/jci.insight.184491DS1). This same macaque also had an incomplete blockade of α4β7 integrin during antibody administration prior to and during ATI (Supplemental Figure 2).

### Coadministration of IL-21 and anti-α4β7 is associated with higher CD4 T cell count and reduced upregulation of PD-1 expression on diverse T cell subsets after ATI.

**Figure 3:**
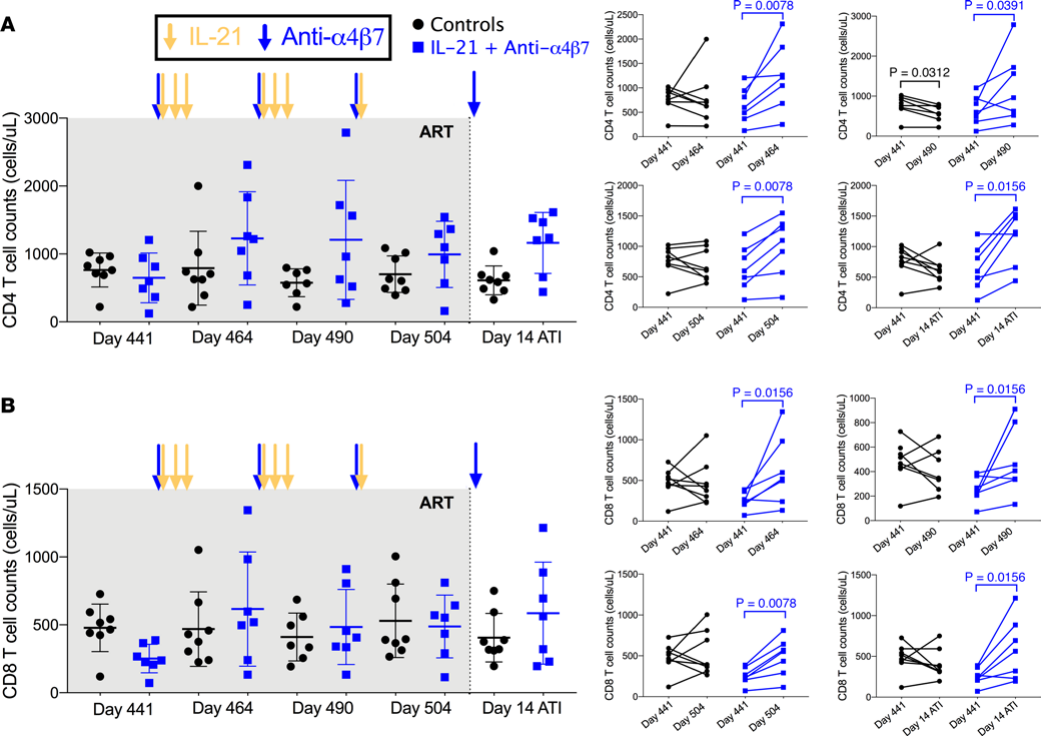
Peripheral CD4^+^ and CD8^+^ T cell counts. (**A** and **B**) Peripheral CD4^+^ (**A**) and CD8^+^ (**B**) T cell counts at days 441, 464, 490, 504, and day 14 after ATI. Data were analyzed by Wilcoxon signed-rank tests.

Individual administration of IL-21 or anti-α4β7 in SIV-infected NHPs significantly modulates peripheral immune dynamics (4, 15). To investigate their influence on peripheral CD4^+^ and CD8^+^ T cells, absolute cell counts were determined for each lymphocyte subset (Figure 3). Dual therapy was associated with a rapid and persistent increase in CD4^+^ T cell counts, which significantly increased as compared with day 441 at day 464 (geometric mean from 533 to 1,023 cells/μL, *P* = 0.0078), day 490 (940 cells/μL, *P* = 0.0391), and day 505 (820 cells/μL, *P* = 0.0078) (Figure 3A). Importantly, this increase was maintained following ATI (day 14 after ATI) with 1,065 cells/μL (*P* = 0.0155). In sharp contrast, control RMs showed decreased CD4^+^ T cell counts at each following time point when compared with day 441, with this decrease reaching significance at day 490 (705–535 cells/μL, *P* = 0.0312) (Figure 3A). Similar increases were found in the dual-treated RMs for CD8^+^ T cells, with their absolute count increasing from 226 cells/μL at day 441 to 487 cells/μL at day 464 (*P* = 0.0156), 413 cells/μL at day 490 (*P* = 0.0156), 421 cells/μL at day 504 (*P* = 0.0078), and 481 cells/μL at day 14 after ATI (*P* = 0.0156) (Figure 3B). No differences were seen in CD8^+^ T cells in controls (Figure 3B). Thus, dual treatment improves the reconstitution of the CD4 and CD8 T cell compartment.

**Figure 4:**
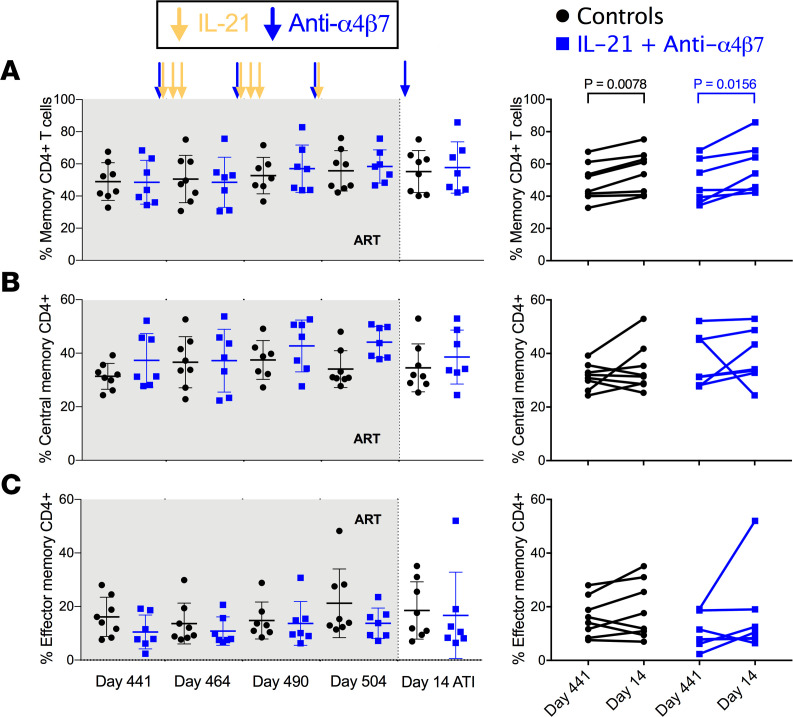
CD4^+^ Memory T cell dynamics. (**A**–**C**) Kinetic changes were measured by flow cytometry determining memory (**A**), CCR7^+^ central memory (**B**), and CCR7^–^ effector memory (**C**) percent T cells in isolated peripheral blood mononuclear cells. Data were analyzed by Wilcoxon signed-rank tests.

To further elucidate the effect of dual therapy on lymphocyte subsets, we first determined by flow cytometry the frequencies of CD4^+^ memory T cell subsets in PBMCs. The frequency of memory (CD95^+^) CD4^+^ T cells was similarly and significantly increased both in control RMs (geometric mean from 47.8% at day 441 to 53.9% at day 14 after ATI [day 518 p.i.], *P* = 0.0078) and in the dual-treated RMs (from 47.0% to 56.0%, *P* = 0.0156) (Figure 4A). Next, CCR7 expression was used to determine the percent of central (TCM, CCR7^+^) and effector (TEM, CCR7^–^) memory T cell subsets longitudinally. There was no significant difference between treatment groups or before and after therapy within either group (Figure 4, B and C).

**Figure 5:**
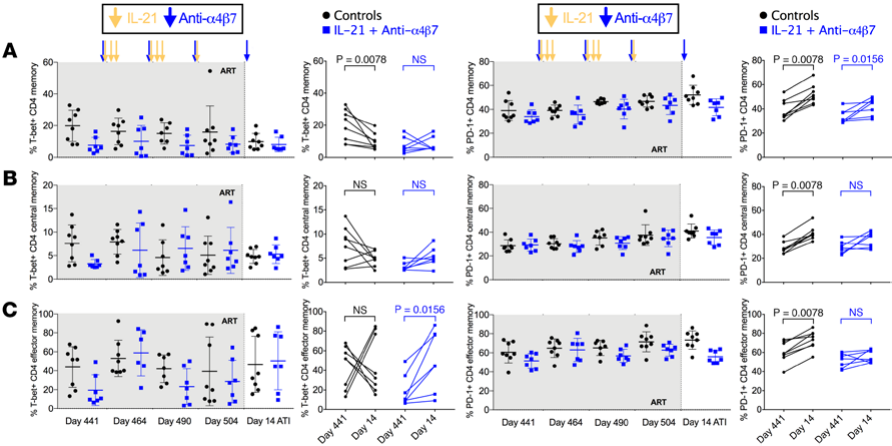
Dual therapy stabilizes T-bet and PD-1 expression on CD4^+^ T cell subsets. (**A**–**C**) T-bet (left) and PD-1 (right) expression was determined longitudinally and compared between predual therapy and day 14 after ATI (day 528 p.i.) for CD4^+^ memory (**A**), CD4^+^ CCR7^+^ central memory (**B**), and CD4^+^ CCR7^–^ effector memory T cells (**C**). Data were analyzed by Wilcoxon signed-rank tests.

T-bet is increased in elite controllers compared with noncontroller PLWH and is required for Th1 differentiation (25, 26). Given the role of IL-21 in driving T-bet expression, we longitudinally evaluated the frequency of T cells expressing T-bet (27). T-bet was significantly reduced on CD4^+^ memory cells at day 14 after ATI in the control but not in the treated animals when compared with day 441 p.i. — i.e., before dual therapy initiation (17.4%–8.8%, *P* = 0.0078, versus 6.4%–7.2%, nonsignificant [NS], respectively) (Figure 5A). Furthermore, T-bet expression in the TEMs, but not in TCMs (Figure 5B), was lower during ART and significantly increased following ATI in the dual-treated group (15.0%–39.3%, *P* = 0.0156) but not in controls (38.0%–38.6%, NS) (Figure 5C). Thus, the frequency of TEM cells expressing T-bet remains stable in untreated controls but significantly increases in the dual-treated RMs. Similar trends were seen in CD8^+^ T cells, with total CD8^+^, CD8^+^ memory, and CD8^+^ TCMs showing significantly lower T-bet expression at day 14 after ATI as compared with day 441 p.i. in the control group and an opposite, NS increase in T-bet expression in CD8^+^ T cell subsets in the dual-treated group (Supplemental Figure 3). Despite these changes, the frequencies of other lymphocyte subsets were not consistently different between groups (Supplemental Figure 4).

The expression of PD-1 on T cells has been associated with both immune exhaustion and viral reservoir establishment (28) and is negatively regulated by T-bet expression (29). PD-1 was significantly increased in the control group at day 14 after ATI as compared with day 441 p.i., including in CD4^+^ memory T cells (from 38.3% to 51.6%, *P* = 0.0078), TCMs (from 28.2% to 40.7%, *P* = 0.0078), and TEMs (from 59.5% to 72.7%, *P* = 0.0078) (Figure 5). Notably, dual-treated animals showed more limited PD-1 upregulation at day 14 after ATI as compared with day 441 p.i. on CD4^+^ memory T cells (from 33.6% to 41.2%, *P* = 0.0156) (Figure 5A), with no significant upregulation neither for TCMs (from 28.5% to 35.1%) nor for TEMs (from 51.0% to 55.6%) (Figure 5, B and C). As a result, the frequency of CD4^+^ TEM cells expressing PD-1 at day 14 after ATI was significantly higher in controls than dual-treated RMs (72.7% versus 55.6%, *P* = 0.0022) (Figure 5C). Similarly to T-bet expression, we found a significant increase in PD-1 expression at day 14 after ATI compared with day 441 in control RMs on total, memory, and effector memory CD8^+^ T cell subsets (Supplemental Figure 3). In contrast, PD-1 expression remained stable, without significant differences between the 2 time points in the dual-treated RMs. Thus, although they had similar levels of PD-1 expression during ART, dual-treated RMs maintained lower levels of PD-1 expression compared with controls at day 14 after ATI. This finding suggests that IL-21+ anti-α4β7 may be reducing T cell activation/inflammation, despite plasma VLS after ATI VLs being comparable among the 2 groups at this time point.

Additional immunophenotyping was performed to further characterize the effect of the dual treatment on peripheral CD4^+^ T cell subsets. These included determining the frequency of Tregs (CD25^+^CD127^–^FOXP3^+^), which were significantly reduced in dual-treated RMs, as compared with day 441, at both day 504 (3.8%–2.2%, *P* = 0.0156) and day 14 after ATI (3.8%–1.4%, *P* = 0.0078) (Supplemental Figure 5, A–C; no difference in controls). Additionally, we quantified CCR6^+^ memory cells (20.4%–27.9%, *P* = 0.0156) and percent of CCR6^+^ memory of total CD4^+^ T cells (10.5%–17.2%, *P* = 0.0156), which both showed a significant increase at day 14 after ATI in dual-treated animals (Supplemental Figure 5, E and F; no difference in controls). RORγt MFI was significantly lower on memory CD4^+^ (7,437–5,299, *P* = 0.0156) and memory CD4^+^CCR6^+^ (7,757–5,681, *P* = 0.0156) T cells at day 14 after ATI as compared with day 441 (Supplemental Figure 5, G and H; no difference in controls). Finally, the MFI of GATA3 on memory CD4^+^ T cells was not different between groups or time points (Supplemental Figure 5, I and J).

### The size of the intact viral reservoirs is associated with PD-1 expression on CD4^+^ T cells and VL rebound after ATI.

**Figure 6:**
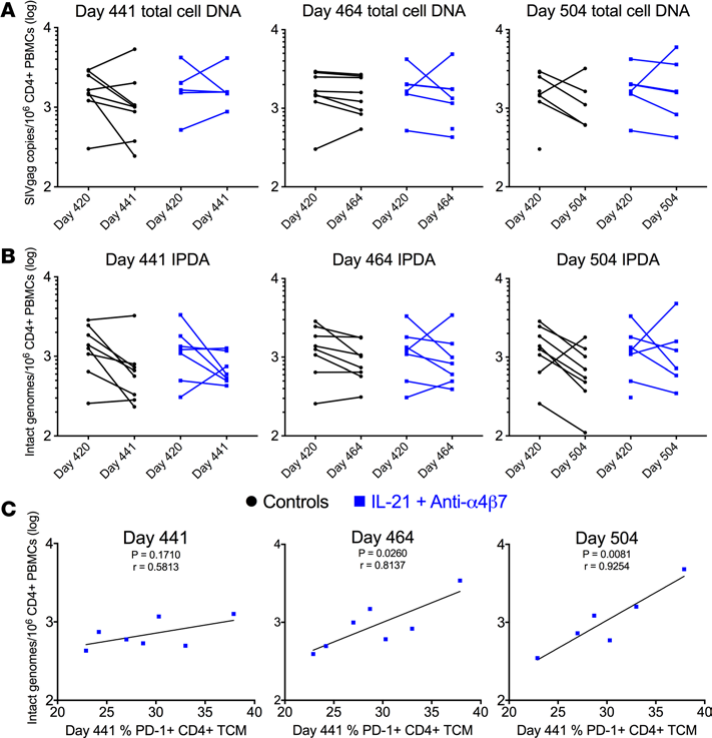
PD-1 expression on CD4^+^ TCMs prior to dual therapy predicts peripheral viral reservoir. (**A** and **B**) CD4^+^ cells were isolated from PBMCs from days 420, 441, 464, and 504 p.i. DNA was isolated, SIVgag copies/1 × 10^6^ cells were determined (**A**), and IPDA was performed for each time point (**B**). (**C**) Linear regression analysis demonstrates PD-1 expression on CCR7^+^ memory cells prior to dual therapy (day 440 p.i.) was associated with intact viral genomes/ 1 × 10^6^ cells in the peripheral CD4^+^ cells and predictive of subsequent measures over the course of treatment.

To determine if dual therapy reduced the viral reservoir, we measured total SIV DNA and intact proviral SIV DNA (IPDA) levels in CD4^+^ T cells from PBMCs at day 420, day 441, day 464, and day 504 (on ART during treatment). Total SIV DNA levels (Figure 6A) or intact SIV DNA levels (Figure 6B) were not significantly different between controls and treated animals at any measured time points. Given previous associations of PD-1 expression with viral reservoir establishment, we evaluated the relationship between PD-1 expression on TCMs and the size of the viral reservoir (Figure 6C). The frequencies of PD-1^+^ TCMs at day 441 were predictive of IPDA measures following therapy initiation at day 464 (*r* = 0.8137, *P* = 0.0260) and day 504 (*r* = 0.9254, *P* = 0.0081) in the dual-treated group but not for control animals. In addition to peripheral CD4^+^ cells, IPDA (Supplemental Figure 6) and CD4^+^ immunophenotyping (Supplemental Figure 7) were also performed for CD4^+^ LN cells, and no significant differences were observed between controls and treated RMs. Thus, a dual IL-21 treatment and blockade of α4β7 does not limit the persistence of the SIV reservoir in blood or LN of ART-treated RMs in this study design.

**Figure 7:**
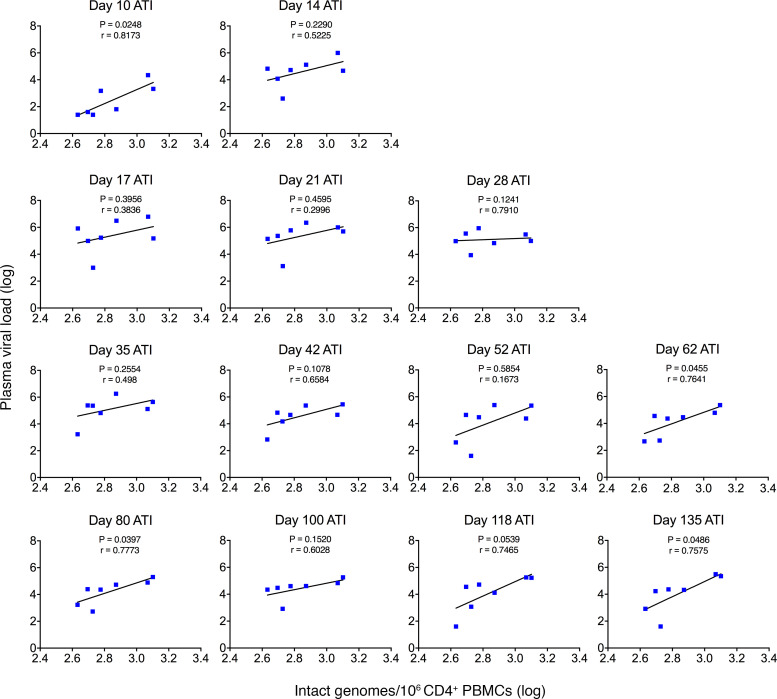
Peripheral CD4^+^ reservoir predicts rebound plasma viral loads. Linear regression analysis was used to compare day 441 p.i. IPDA with rebound viral loads following dual therapy.

Next, we assessed whether the intact viral reservoir size before therapy initiation predicted viral rebound after ATI. These 2 measures were significantly associated at multiple ATI time points, including at day 10 after ATI (*r* = 0.8173, *P* = 0.0248), at day 62 after ATI (*r* = 0.7641, *P* = 0.0455), and at day 135 after ATI (*r* = 0.7575, *P* = 0.0486) (Figure 7). Again, these associations were only present in the dual-therapy group and not in controls.

### Dual therapy facilitates recovery of fecal microbiome composition.

**Figure 8:**
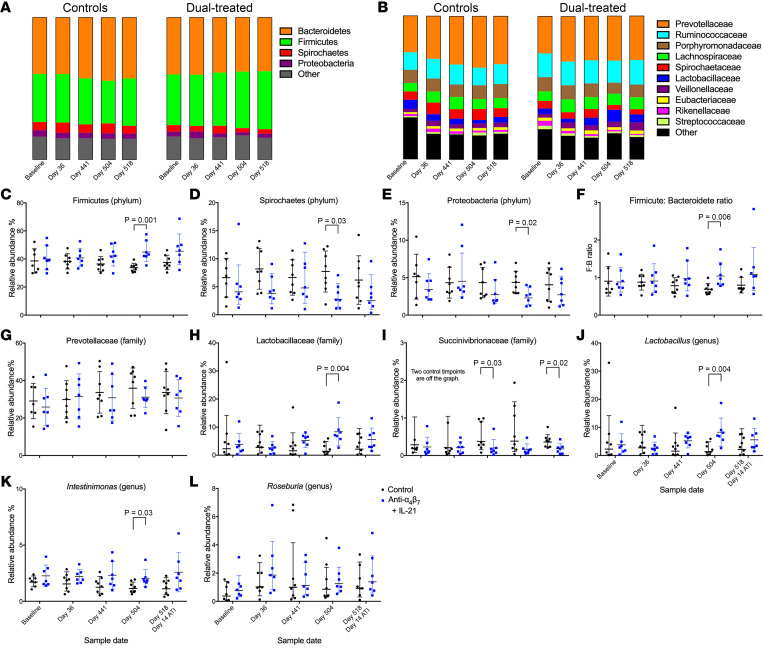
16S rRNA Fecal Microbiome Sequencing. (**A** and **B**) Bar graphs illustrating the relative abundance of bacteria phyla (**A**) and families (**B**). (**C**–**L**) Longitudinal comparisons between control with dual-treated RMs including the phyla Firmicutes (**C**), Spirochaetes (**D**), and Proteobacteria (**E**); the Firmicute/Bacteroidete ratio (**F**); the families Prevotellaceae (**G**), Lactobacillaceae (**H**), and Succinivibrionaceae (**I**); and the genera *Lactobacillus* (**J**), *Intestinimonas* (**K**), and *Roseburia* (**L**). Each taxon was analyzed by Mann-Whitney *U* tests comparing control with dual-treated RMs at each time point.

HIV and SIV are associated with acute- and chronic-infection–associated dysbiosis of the fecal microbiome. Because both IL-21 and anti-α4β7 therapy individually affect intestinal mucosal immune response, we next performed 16S rRNA gene sequencing on longitudinal fecal samples to characterize changes in microbial communities during both infection and therapeutic intervention (9, 22, 30). Differential changes in the relative abundance of phyla and families are summarized in stacked bar graphs (Figure 8, A and B). This includes a higher relative abundance of Firmicutes (44.9% versus 33.7%; *P* = 0.001) at day 504 following dual-therapy administration in treated RMs compared with controls (Figure 8C). Additionally, the relative abundance of Spirochaetes (6.7% versus 2.8%; *P* = 0.03) and Proteobacteria (4.2% versus 2.3%, *P* = 0.02) were significantly higher in controls compared with the dual-treated group at day 504, the latter of which is a characteristic of HIV/SIV-associated dysbiosis (Figure 8, D and E) (31, 32). We then calculated the Firmicute/Bacteroidete (F:B) ratio, a microbiome marker typically reduced during gut inflammation and IBDs (33–35). Notably, the F:B ratio was significantly higher in dual-treated animals than in controls at day 504 (1.04 versus 0.68; *P* = 0.006) (Figure 8F). However, no significant differences were seen in the family Prevotellaceae (Figure 8G). The 5 most abundant Firmicute families (Figure 8B), which included Ruminococcaceae (14.8% versus 11.8%), Lachnospiraceae (7.6% versus 6.5%), Lactobacillaceae (7.2% versus 1.4%), Veillonellaceae (3.5% versus 2.8%), and Eubacteraceae (1.6% versus 1.5%), all trended toward higher frequencies in the dual-treated RMs than controls, with only Lactobacillaceae being statistically significant (*P* = 0.004) (Figure 8, B and H). Additionally, the less abundant Succinivibrionaceae, a member of Proteobacteria, was significantly lower in the dual-treated group at 520 (*P* = 0.02) (Figure 8I). Of the top 50 genera analyzed, 2 were statistically different between groups at day 504, including *Lactobacillus* (*P* = 0.004) (Figure 8J) and *Intestinimonas* (*P* = 0.03) (Figure 8K). The overall differences between the control and dual-treated animals suggest that the combination strategy utilized in this study can partially restore eubiosis following SIV-associated dysbiosis. Finally, to determine if the described changes in microbiome composition directly affected barrier function, we longitudinally quantified by ELISA plasma levels of LPS and soluble suppression of tumorigenicity 2 (sST2) , markers used to evaluate barrier integrity during HIV (36, 37). No statistical difference was found between groups at measured time points (Supplemental Figure 8).

### The relative abundance of the commensal bacteria Roseburia is predictive of SIV progression markers following dual-therapy administration.

**Figure 9:**
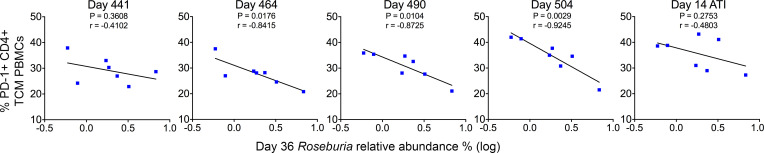
The relative abundance of *Roseburia* at acute infection is negatively associated with PD-1 expression on CD4^+^ TCMs and predictive of subsequent PD-1 expression. Linear regression analysis comparing the relative abundance of *Roseburia* at day 36 p.i. with PD-1 expression on CD4^+^CCR7^+^ central memory T cells isolated from peripheral blood.

Previous studies have reported that the relative abundance of *Roseburia* is associated with immunological outcomes in PLWH (38). *Roseburia* has recently been shown to be a determinant of anti–PD-1 therapeutic efficacy against solid tumors in murine models. On its own, *Roseburia* supplementation outperformed anti–PD-1 monotherapy in reducing tumor size after administration (39). To determine if there was a molecular link between *Roseburia* and PD-1 expression in our model, linear regression was performed between the relative abundance of *Roseburia* (Figure 8L) and PD-1 expression on TCMs, a CD4^+^ cell type thought to constitute the majority of the long-lived reservoir, but not TEMs (40, 41). All time points of microbiome sampling were compared. Still, only day 36 (the time point nearest to acute infection and associated dysbiosis) was predictive of subsequent changes in the percent PD-1^+^ TCMs following therapy initiation at all time points prior to ATI, including days 464 (*r* = –0.8415, *P* = 0.0176), 490 (*r* = –0.8725, *P* = 0.0104), and 504 (*r* = –0.9245, *P* = 0029) (Figure 9), a finding consistent with baseline abundance of *Roseburia*, but not later time points, being predictive of vedolizumab (anti-α4β7) efficacy for IBD (30).

**Figure 10:**
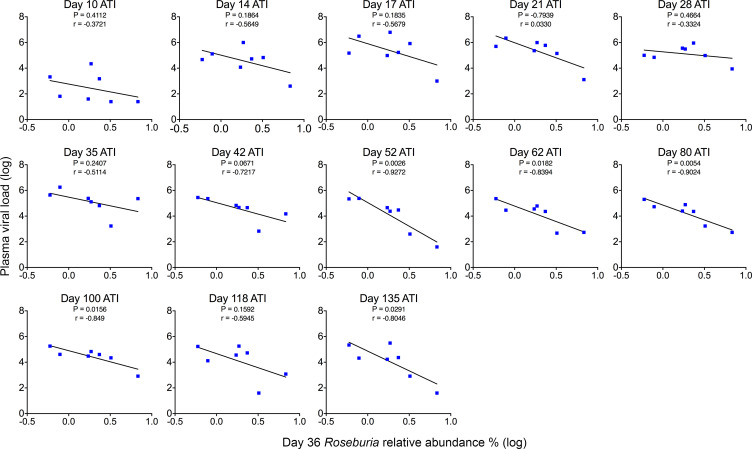
The relative abundance of *Roseburia* at acute infection is predictive of rebound plasma viral load in dual-treated RMs. Linear regression analysis was utilized to compare day 36 p.i. relative abundance of *Roseburia* with subsequent plasma viral loads following ATI in dual-treated RMs. Statistical significance began at day 52 ATI.

**Figure 11:**
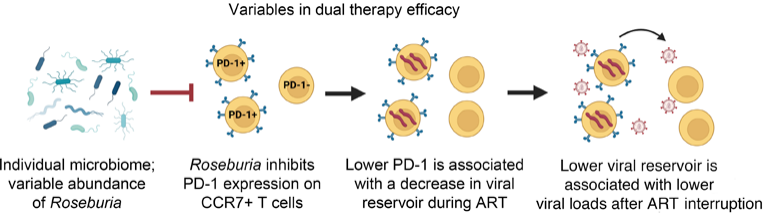
Variables in dual therapy efficacy. The proposed strategy of dual therapeutic administration of anti-α4β7 mAb with IL-21–Fc following viral suppression of ART was associated with a significant decrease in rebound plasma viral load following ATI compared with viral set point. We identified 3 key interrelated variables associated with improved control. *Roseburia* abundance in the fecal microbiome was negatively associated with PD-1 expression on CCR7^+^ memory CD4^+^ T cells. PD-1 expression was then negatively associated with predual therapy peripheral viral reservoir size, which was predictive rebound viral load. Finally, day 36 p.i. *Roseburia* relative abundance was negatively associated with viral loads beginning on day 52 ATI suggesting microbiome composition was a determinant of efficacy.

We next tested whether the day 36 relative abundance of *Roseburia* was also predictive of VL during ATI. Using linear regression analysis, we found that similarly to day 441 IPDA measures, the day 36 relative abundance of *Roseburia* was predictive of VL following ATI. As previously noted, rebound VLs were initially higher in the dual-treated group than in controls and only fell below controls at day 42. Only after this time point does day 36 *Roseburia* relative abundance become predictive of VLs (*r* = –0.9272, *P* = 0.0026) (Figure 10). This association was maintained at most time points (including days 52, 62, 80, 100, 135) until the last point at day 135 (*r* = –0.8046, *P* = 0.0291). Overall, although unable to affect reservoir size and time to viral rebound after ATI, the IL-21 and anti-α4β7 strategy described here promoted recoveries from acute SIV-associated dysbiosis, limited PD-1 expression on several immune cell subsets, and slightly reduced VLs in the absence of ART. Based on these factors, we propose a model of interrelated variables that contribute to the efficacy of our dual-therapy strategy (Figure 11).

## Discussion

Despite the effectiveness of ART, there is currently no cure for HIV. Here we tested what we believe to be a novel dual-therapy strategy combining anti-α4β7 and IL-21 during suppressive ART, 2 therapies that modify mucosal immune responses and have shown promising early results as monotherapies (4, 15). This strategy significantly increased CD4^+^ and CD8^+^ T cells in the periphery and slightly improved virologic control in the absence of ART, with dual-treated RMs maintaining a VL significantly lower than the set point for the ATI follow-up, unlike the control animals. This rebound VL was sequentially associated with several disease markers, including viral reservoir, PD-1^+^ TCMs, and the relative abundance of *Roseburia*. We had to exclude 1 dual-treated RM from the analysis due to incomplete ART suppression prior to ATI. This RM, which did not show an improvement in viral control after dual therapy, had higher baseline dysbiosis and incomplete α4β7 masking prior to and during ATI. The findings on this excluded animal align with our model, identifying several predictors of dual-therapy efficacy, including baseline microbiome composition, PD-1 expression on long-lived TCMs, and associated viral reservoir.

Because 2 therapies were utilized prior to ATI, it is impossible to determine the relative contribution of each to VL rebound. Previously, IL-21 monotherapy slightly reduced VL rebound following ATI (4). Additionally, IL-21 can improve Th17 reconstitution and intestinal barrier function in SIV-infected PMs when coadministered with a probiotic cocktail containing *Lactobacillus* (9). We found dual therapy was associated with a significant increase in *Lactobacillus* compared with controls, but whether this increase played a role in efficacy requires additional investigation. On the other hand, reports on anti-α4β7 have been mixed (15, 18–21). In a recent study, administering anti-α4β7 in conjunction with broadly neutralizing antibodies (bNAbs) significantly increased the time to viral rebound compared with bNAbs alone in simian-human immunodeficiency virus–infected (SHIV_SF162P3_-infected) macaques (18). In another study, administering anti-α4β7 to ART-suppressed SIVmac239-nef-stop–infected. RMs facilitated persistent virologic control during ATI (15). Notably, subsequent RM studies did not replicate viral control, suggesting that further factors such as host genetics and microbiome composition may also contribute to the therapy’s efficacy (18–22). Although the dual-treated RMs in the present study did not experience viral control, they showed significant increases in CD4^+^ T cell counts, including increased CCR6^+^ memory CD4^+^ T cells and significant decreases in memory CD4^+^ Tregs, each consistent with this previous study (15). Notably, the accessory protein Nef is necessary and sufficient for PD-1 expression on human PBMCs ex vivo and can induce expression independent of chronic immune activation (42). If, as our data suggest, PD-1^+^ expression on TCMs is a key hurdle to anti-α4β7 efficacy, reduced PD-1 expression (although not quantified in the SIVmac239-nef-stop study) may contribute to the discrepancies between the prior research with a nef-stop virus and the data we present herein (15). This hypothesis would be consistent with a recent clinical trial in which people with recently acquired HIV were administered vedolizumab concurrently with ART (43). These individuals experienced a significantly longer time to first VL > 1,000 copies/mL following ATI than historical controls. However, cell-associated HIV DNA persisted in PBMCs and, similar to the findings in our study, was positively associated with the frequency of PD-1^+^ memory CD4^+^ T cells (43).

Exogenous IL-21 and anti-α4β7 mAb were administered to SIV-infected, ART-treated RMs to recapitulate critical features of nonprogressive SIV infections in SMs (10, 14). Besides differences in IL-21 production and α4β7 integrin expression by lymphocytes, SMs have lower CCR5 expression on TCMs than RMs. We submit that these differences contribute to SM TCMs resistant to infection and subsequent limited reservoir seeding (44), providing a mechanism for increased TCM infection rates in RMs compared with SMs (45). In addition to reduced CCR5 expression, SMs upregulate PD-1 on T cells early during infection to reduce immune activation, while RMs upregulate PD-1 later following infection (46). The combination of late PD-1 expression and increased infectivity of TCMs in RMs may explain why PD-1 expression on TCMs was predictive of viral reservoir size in our study. Furthermore, TCM infection is associated with plasma VLs in RMs but not in SMs (45), a finding consistent with our model in which PD-1^+^ TCMs are predictive of VL rebound after ATI in addition to the size of the viral reservoir.

Similar to the comparison between a progressive (RMs) and a nonprogressive (SMs) model for SIV infection, there are critical differences in PLWH who experience disease progression as compared with those more resistant to disease (47). Firstly, nonprogressing PLWH have significantly reduced TCM infection compared with progressors (48). Secondly, the preservation of TCMs after long-term ART has been associated with improved odds of posttreatment virological remission after ATI (49). Finally, 2 MHC-I alleles, HLA-B*27 and HLA-B*57, are related to long-term nonprogressor status. These alleles are associated with preserving the TCM compartment from infection, which subsequently is associated with improved preservation of TCM counts (50). These findings point to TCMs as a specific component of virologic control or nonprogressor status in viremic individuals. In addition to the role of TCMs in HIV infection, PD-1^+^CD4^+^ T cells have been correlated with the levels of proviral reservoir and are associated with reduced total CD4^+^ T cell counts in humans (51). Additionally, PD-1^hi^ TCMs are enriched for the presence of provirus compared with PD-1^lo^ TCMs in PLWH (40). Thus, PD-1 expression on TCMs as a primary variable in viral control in our model after ATI is consistent with evidence generated by comparing progressive and nonprogressive HIV and SIV infections in humans and NHP.

HIV infection and IBDs are each associated with a significant reduction in levels of butyrate-producing Firmicute *Roseburia* (38, 52). Recent research has indicated that the relative abundance of *Roseburia* is predictive of vedolizumab efficacy in patients with IBD (30). Additionally, our team recently found that the ratio of *Prevotella*/*Roseburia* was associated with increased gut lamina propria macrophage maturity in anti-α4β7–treated RMs (22). This finding was built on previous work in PLWH, suggesting that *Roseburia* and other butyrate-producing bacteria are associated with immunological outcomes of PLWH on ART (38). Despite these findings, administering sodium butyrate as adjunctive therapy to ART in SIV-infected RMs does not significantly improve disease outcomes (53). This result may be due to butyrate’s propensity to inhibit intestinal stem cell proliferation, even though butyrate serves as the primary energy source for mature colonocytes, a phenomenon known as the butyrate paradox (54, 55). This explanation is consistent with additional findings that butyrate supplementation was associated with NS increases in plasma IFABP2, sCD14, and translocation index measurements in SIV-infected, ART-suppressed RMs compared with controls (53). *Roseburia* has been associated with reduced solid tumor burden in murine colon cancer models, acting synergistically with anti–PD-1 mAb and performing better than anti–PD-1 alone (39). However, these findings were independent of fecal or serum butyrate levels (39). As noted in the SIV-infection model, sodium butyrate supplementation did not provide immunological benefit in other murine cancer models, and its administration reduced the efficacy of anti-CTLA-4 therapy in these studies (56). Thus, *Roseburia* may influence immune response independent of its butyrate-producing capabilities. This conclusion is supported by our recent finding that *Roseburia* was not strongly correlated with fecal butyrate in SIV-infected RMs (57). Additionally, *Roseburia* is not unique in its ability to produce butyrate. Instead, *Roseburia*’*s* unique “silent” flagellin, which can bind to TLR5 without activating it, may be inhibiting gut inflammation, with or without butyrate (58–60).

Herein, we present that *Roseburia* abundance is negatively associated with subsequent PD-1 expression on TCMs and their establishment as long-lived cellular reservoirs. Notably, this association was found only with *Roseburia* abundance during acute infection, the time at which persistent HIV reservoirs are thought to be established (61). However, *Roseburia* also significantly influences IL-10 signaling and regulation of T cell function by CTLA-4 in mouse models, so we cannot rule out additional pathways that we have not examined in this study but that we have previously described in reservoir establishment (62–64). These findings suggest that the relative abundance of *Roseburia*, rather than butyrate alone or the presence of other butyrate-producing bacteria, is crucial for the efficacy of anti-α4β7 therapy during SIV infection. This relationship is important in reducing potential viral reservoirs and promoting subsequent viral control. Thus, efforts to modulate microbiome composition during HIV/SIV, such as fecal microbiota transplants (FMT) or prebiotic supplementation, are warranted. Clinical trials have demonstrated successful engraftment of *Roseburia* in patients with ulcerative colitis, which was associated with achieving remission (65). Similar FMT pilot trials have been performed for PLWH (66, 67). When FMTs were performed in SIV-infected RMs, successful engraftment of *Roseburia* was associated with reduced markers of CD4^+^ T cell activation in the gut (68). Other efforts to influence microbiome composition, such as prebiotics or other dietary supplements, may also have a place in improving clinical response to mucosal immune responses (9). For example, polyphenol-rich berry camu-camu improves antitumor response during anti–PD-1 therapy in mice by modulating microbiome composition (69, 70).

PD-1 expression on TCMs was a unique predictor of viral reservoir size and rebound VL only in the dual-treated macaques, and a recent clinical trial similarly found PD-1 expression on memory cells to be associated with HIV copies in cell-associated DNA following vedolizumab therapy with ART (43). These findings suggest that PD-1^+^ memory cells, and perhaps PD-1^+^ TCMs specifically, remain a crucial hurdle in viral control following anti-α4β7 administration. This limited cell population may therefore offer an adjunctive therapeutic target. Under its role in suppressing cell activation, the expression of PD-1 has a dual role in aiding viral reservoir formation by promoting CD4^+^ T cell latency and reducing T cell effector function (28). In addition to the *Roseburia* abundance discussed above, therapies specifically designed to block the PD-1/PD-L1 pathway already exist and have been utilized in both SIV infection models and clinical trials for HIV, with varying levels of success (71–76). Importantly, vedolizumab is an effective intervention for immune checkpoint inhibitor–induced (CPI-induced) colitis, a major concern of CPI due to increased T cell activation, suggesting that the coadministration may provide a reciprocal benefit absent from current anti–PD-1 monotherapy (77). However, further studies are needed, as disruption to the α4β7/MAdCAM-1 axis has been associated with reduced anti–PD-1 efficacy during cancer therapy (78). Interestingly, this study also found specific microbial taxa, including *Enterocloster*, that can downregulate MAdCAM-1 expression, an interaction not examined in our study (78). One limitation of this study is that CTLA-4 expression was not determined on T cell subsets. However, like PD-1, CTLA-4 is modulated by *Roseburia* abundance and acts as an immune checkpoint (62). CTLA-4 expression may, therefore, also be a factor in promoting reservoir persistence, as we have previously described, and should be examined in more detail in future studies (64). As for PD-1, anti–CTLA-4 mAbs are also available and used in HIV/SIV trials (71, 79–82). Finally, in addition to the utilization of CPIs, a better understanding of the role of *Roseburia* in the microbiome beyond butyrate production is vital for harnessing its potential in modulating HIV pathogenesis and immune function in other disease states such as IBDs and cancer. Understanding these remaining barriers to virologic control is critical to improving the design of combined immune-modulating therapies aimed at curing HIV.

## Methods

### Sex as a biological variable.

Our study utilized both male and female animals, and similar findings are reported for both sexes.

### Study design.

Indian-origin RMs (*n* = 16) (obtained from ENPRC breeding colony) were infected with 300 TCID_50_ SIVmac239 acquired from Koen Van Rompay at UCD (Davis, California, USA). Beginning on day 42 (week 6) p.i., ART (tenofovir disoproxil fumarate [TDF], 5.1 mg/kg/d, Gilead Sciences; emtricitabine [FTC], 40 mg/kg/d, Gilead Sciences; and dolutegravir [DTG], 2.5 mg/kg/d, ViiV Healthcare) was administered daily until day 503 (for a total of 72 weeks) followed by ART interruption. RMs were stratified into 2 treatment groups (*n* = 8, each; 1:1 ratio) based on acute (“peak”) and set point (“chronic” at ART initiation) VL; duration of virologic suppression; MHC haplotype (*Mamu*-A*01^+^); sex; and age. Beginning on day 448 (week 64) p.i., the experimental group was administered 50 mg/kg anti-α4β7 mAb (obtained from NIH Nonhuman Primate Reagent Resource, University of Massachusetts Medical School) i.v. every 3 weeks until day 574 (week 82) p.i. (day 70 ATI) for a total of 7 administrations. This dosage is higher than vedolizumab administered to patients with IBD but consistent with other NHP studies, in better mask the α4β7 integrin epitope (15, 18). In addition, beginning at day 448 (week 64) p.i., the same experimental group received weekly s.c. administration of 100 μg/kg IL-21–IgFc (8) (obtained from Resource for Nonhuman Primate Immune Reagents of the New Iberia Research Center) and continued until just before ART interruption (day 490; week 70 p.i.; 7 administrations total). An experimental schematic is shown in Figure 1. RM weights were monitored during the dual therapy (Supplemental Figure 9). A summary of RM characteristics can be found in Supplemental Table 1.

### Sample collection and cell isolation.

Blood was collected at varying time points (Figure 1) in ETDA tubes. Plasma was separated and collected. Density gradient centrifugation (800*g* for 30 minutes with the break off) was performed to isolate peripheral blood mononuclear cells (PBMCs) for flow cytometry and characterization of the viral reservoir. Lymph nodes were collected on days 441, 464, 504, and 518, and LN cells were isolated by mincing and filtering tissue with 70 μm cell strainers. Aliquots of fresh cells from each collection event were used for flow cytometry within 24 hours of collection. An additional aliquot of lymphocytes was frozen and used for flow cytometric analysis to further characterize the phenotype and functional subsets of CD4^+^ T cells.

### Flow cytometry.

An aliquot of each isolated PBMC and LN cell sample was stained, fixed (1% paraformaldehyde), and analyzed by standard flow cytometry using protocols and mAbs that were cross-reactive with RM immune cells (Supplemental Table 2). The following antibodies were used: anti–NKG2a-APC (clone NKG2a-APC) from Beckman Coulter; anti–CD4-APCCy7 (clone OKT4), anti–CD20-PerCpCy5.5 (clone 2H7), anti–PD-1–BV785 (clone EH12.2H7), and anti–T-bet–BV421 (clone 4B10), all from BioLegend; anti–CCR7-Cy7PE (clone 3D12), anti–CD3-BUV395 (clone SP34-2), anti–CD8-BUV496 (clone RPA-T8), anti–CD14-BUV805 (clone M5E2), anti–CD28-BUV737 (clone CD28.2), anti–CD56-BV711 (clone B159), anti–CD95-Cy5PE (clone DX2), HLA-DR–BV605 (clone G46-6), and Ki67-BV480 (clone B56), all from Becton-Dickinson, BD Biosciences; anti–CXCR5-PE (clone MU5UBEE) from eBioscience; anti–GranB-PETR (clone GB11) from Invitrogen; anti–CD38-FITC (clone AT1) from STEMCEL Technologies; and anti–α4β7 (A4B7R1)-PE (clone A4B7R1) from NIH Nonhuman Primate Reagent Resource, University of Massachusetts Medical School. Event acquisition was performed on a BD LSRII Flow Cytometer with BD FACSDiva software, and data were collected from at least 100,000 CD3^+^ events. Analysis was then performed using FlowJo software (Tree Star Inc.). A representative gating strategy is displayed in Supplemental Figure 10. Monocyte subsets are reported in Supplemental Figure 11.

A second flow cytometry analysis was performed on PBMCs that were frozen and stored at –150°C. These cells were later thawed and stained with the following antibodies: anti–CD3-BUV395 (clone SP34-2), anti–CD8-BUV496 (clone RPA-T8), anti–CD28-BUV737 (clone CD28.2), and anti–CCR6-BV421 (clone 11A9) from Becton-Dickinson, BD Biosciences; anti–CD4-APC-Cy7 (clone OKT4), anti–CD25-BV711 (clone BC96), anti–CD95-PE-Cy5 (clone DX2), anti–FoxP3 Alexa Fluor 647 (clone 150D) from BioLegend; anti–CD127-Alexa Fluor 488 (clone eBioRDR5), anti–GATA3-BV605 (clone TWAJ), and anti–RORyt-PE (clone AFKJS-9) from Thermo Fisher Scientific; as well as Fixable Viability Stain 700 (Becton-Dickinson, BD Biosciences; catalog 317418) (Supplemental Table 3).

### Plasma VLs quantification.

Frozen plasma samples were sent to the UNMC as well as Quantitative Molecular Diagnostics Core of the AIDS and Cancer Virus Program of the Frederick National Laboratory for determination of VLs by quantitative PCR (qPCR) as previously described (83, 84). Briefly, RNA was isolated using a QIAamp viral RNA Mini Kit (Qiagen, product no. 52906). SIV gag RNA was quantified using TaqMan RNA-to-Ct 1-Step kit (Thermo Fisher Scientific, 4392938) with a QuantStudio 3 real-time PCR system (Applied Biosystems). Beginning at day 441 (the last time point prior to dual therapy initiation), the method of VL determination was switched to a hybrid quantitative real-time/digital PCR. This method increases the precision of qPCR and the accuracy of digital PCR approaches as previously described (85). Briefly, RNA was extracted from plasma and divided into 6 replicates for a 2-step qPCR reaction. If all replicates are positive, an average Ct value was used, but a Poisson determination was utilized if only a fraction were positive. Primers and probes targeting conserved SIVmac239 *gag* incorporating neutral bases (to avoid off-target viral sequences) were used. This method reduces the detectable VL threshold to 7.5 copies/mL (85).

### Cell-associated SIV DNA.

Frozen isolated PBMCs were sent to UNMC on dry ice. CD4^+^ cells were isolated as described (83) using an EasySep NHP CD4^+^ T cell isolation kit from STEMCELL Technologies Canada Inc. (catalog 19582). Isolated cells were then lysed, and DNA was isolated using DNeasy Blood & Tissue Kits (Qiagen, 69504). Isolated DNA was quantified using a SimpliNano spectrophotometer (GE Healthcare Bio-Sciences Corp.). CD4^+^ Cell-Associated DNA was quantified using droplet digital PCR (ddPCR) with 0.5 μg DNA added to 2× ddPCR Supermix for probes (no dUTP) (Bio-Rad; 1863024) with SIVgag-specific primers and probes. In total, 22 μL of the reaction mixture generated droplets onto a ddPCR plate with a QX200 droplet generator. The plate was heat sealed with foil (Bio-Rad; 181-4040), and the reaction mixture was amplified with a C1000 Touch thermal cycler (Bio-Rad). The droplets were then characterized for fluorescence with a QX200 droplet reader (Bio-Rad), and the DNA copies/μL were calculated using QuantaSoft software. These readings were then normalized with copies of the RM RPP30 gene, also determined by ddPCR as previously described, to determine the total number of SIVgag DNA copies per million cells (86).

### Intact proviral DNA assay.

An intact proviral DNA assay (IPDA) was utilized as previously described (87). Briefly, 250–1,000 ng DNA from isolated CD4^+^ T cells was added to a mix of 10 μL 2× ddPCR Supermix for probes (no dUTP) (Bio-Rad; 863024), 600 nM primers, and 200 nM probes. ddPCR was performed as outlined above. The quantification of intact proviral DNA per million cells was determined by normalization with copies of RPP30 gene using ddPCR (86).

### 16S rRNA-Seq.

Fecal samples were collected at baseline and on days 36, 441, 504, and 518; frozen at each time point; and stored at –80°C before analysis. Fecal samples were homogenized with a TissueLyser LT (Qiagen, 69980), and DNA was isolated using spin column chromatography-based Stool DNA Isolation Kit (Norgen Biotek Corp., 27600). Each sample’s V3-V4 16S rRNA variable regions were amplified and indexed to generate a library at the UNMC Genomics Core. Sequencing was performed with an Illumina MiSeq platform and demultiplexed with Illumina Basespace, and FASTQ data files were created. Taxonomic identity was assigned using the Illumina Basespace 16S Metagenomics app, which utilizes a Ribosomal Database Project (RDP) Classifier and DADA2 formatted FASTA from the RefSeq RDP 16S v3 database. FASTQ samples were registered and accessible through the NCBI BioProject ID: PRJNA1117186.

### ELISAs.

LPS and sST2 assays were conducted by the Biomarkers Core at the ENPRC, using commercially available ELISA kits validated for rhesus plasma samples. All plasma samples and reagents were thawed to room temperature prior to assay. For LPS measurement, plasma samples were diluted 1:10 using endotoxin-free water and heat treated at 70°C for 10 minutes to inactivate plasma proteins (37). Samples in duplicate, standards, and controls were assayed according to the ELISA kit manufacturer’s instructions (LSBio, F17912). Plasma samples for sST2 determination were analyzed undiluted in duplicate, along with standards and controls, according to the ELISA kit manufacturer’s instructions (LSBio, F57077). Data analyses were done using SoftMax PRO software 7.0. All data were analyzed at 450 nm using 4 parameter logistic (4-PL) curve-fit. The intra- and interassay CV values were 13.6% and 18.5% for the LPS assay and 6.8% and 12.0% for the sST2 assay, respectively.

### Statistics.

GraphPad PRISM (GraphPad Software Inc.) was used for all statistical analyses. Ratio paired 2-tailed *t* tests were utilized when comparing longitudinal VLs. Gaussian distribution for these data was confirmed using Shapiro-Wilk normality tests of the ratio of each time point VL compared with the same RM’s respective set point VL (day 42), following log_10_ transformation. All groups were normally distributed at each time point, except day 118 ATI, when 1 RM in the dual-treated group controlled the virus better than others (i.e., its ratio was outside normal distribution). *P* < 0.001, regardless of whether it was treated as an outlier, and the difference for this comparison remained significant when tested by the Wilcoxon signed-rank test (*P* = 0.0156). Wilcoxon signed-rank tests were utilized when comparing cell counts and flow cytometry data taken longitudinally, with data paired to the respective RM, and Mann-Whitney *U* tests were used when comparing between groups. Mann-Whitney *U* tests were used to compare microbiome relative abundances between groups at a given time point. Linear regression analysis was used to determine correlations and strength of fit (*r*). *P* values less than 0.05 were considered significant. One macaque (14_13) in the treated group was excluded from statistical analysis between groups because it experienced ART failure prior to ATI.

### Study approval.

This study was approved by the IUCAC (no. 2002876) at the Emory National Primate Center. All animal experiments complied with *Guide for the Care and Use of Laboratory Animals* (National Academies Press, 2011). All procedures were performed per institutional regulations and following approval by the IUCAC (no. 2002876) at the Emory National Primate Center in Atlanta, Gerogia, USA. Animal care facilities are accredited by the U.S. Department of Agriculture (USDA) and the Association for Assessment and Accreditation of Laboratory Animal Care (AAALAC) International.

### Data availability.

16S rRNA-Seq FASTQ files are available on the NCBI BioProject database with the BioProject ID: PRJNA1117186. Values for all data points in graphs are reported in the Supporting Data Values file.

## Author contributions

M Paiardini and SNB designed the study. M Pino led the in vivo study scheduling, specimen processing, and flow cytometry analysis. JAC performed additional flow cytometry analysis during revision. AA quantified plasma VLs and performed reservoir assays. DB performed IgA antibody response assays. SDJ generated FASTQ files and performed 16S metagenomics. SDJ and M Pino performed data analysis and wrote the manuscript draft. FV provided IL-21–Fc and edited the manuscript. M Paiardini and SNB acquired funding, oversaw the study, and edited the manuscript. KN and JH contributed to specimen processing, flow cytometry acquisition, and analyses. All authors contributed to manuscript development and have critically reviewed and approved the final version.

## Supplementary Material

Supplemental data

Supporting data values

## Version 1 02/04/2025

In-Press Preview

## Version 2 03/24/2025

Electronic publication

## References

[1] https://www.unaids.org/en/resources/documents/2022/in-danger-global-aids-update.

[2] Sabin CA (2013). Do people with HIV infection have a normal life expectancy in the era of combination antiretroviral therapy?. BMC Med.

[3] Thurman M (2020). Biomarkers of activation and inflammation to track disparity in chronological and physiological age of people living with HIV on combination antiretroviral therapy. Front Immunol.

[4] Micci L (2015). Interleukin-21 combined with ART reduces inflammation and viral reservoir in SIV-infected macaques. J Clin Invest.

[5] Gordon SN (2010). Disruption of intestinal CD4^+^ T cell homeostasis is a key marker of systemic CD4^+^ T cell activation in HIV-infected individuals. J Immunol.

[6] Brenchley JM (2008). Differential Th17 CD4 T-cell depletion in pathogenic and nonpathogenic lentiviral infections. Blood.

[7] Favre D (2009). Critical loss of the balance between Th17 and T regulatory cell populations in pathogenic SIV infection. PLoS Pathog.

[8] Micci L (2012). Paucity of IL-21–producing CD4(+) T cells is associated with Th17 cell depletion in SIV infection of rhesus macaques. Blood.

[9] Ortiz AM (2016). IL-21 and probiotic therapy improve Th17 frequencies, microbial translocation, and microbiome in ARV-treated, SIV-infected macaques. Mucosal Immunol.

[10] Pallikkuth S (2013). Maintenance of intestinal Th17 cells and reduced microbial translocation in SIV-infected rhesus macaques treated with interleukin (IL)-21. PLoS Pathog.

[11] Chevalier MF (2011). HIV-1-specific interleukin-21^+^ CD4^+^ T cell responses contribute to durable viral control through the modulation of HIV-specific CD8^+^ T cell function. J Virol.

[12] Berlin C (1993). Alpha 4 beta 7 integrin mediates lymphocyte binding to the mucosal vascular addressin MAdCAM-1. Cell.

[13] McLean LP (2012). Vedolizumab for the treatment of ulcerative colitis and Crohn’s disease. Immunotherapy.

[14] Byrareddy SN (2015). Species-specific differences in the expression and regulation of α4β7 integrin in various nonhuman primates. J Immunol.

[15] Byrareddy SN (2016). Sustained virologic control in SIV+ macaques after antiretroviral and α4β7 antibody therapy. Science.

[16] Byrareddy SN (2014). Targeting α4β7 integrin reduces mucosal transmission of simian immunodeficiency virus and protects gut-associated lymphoid tissue from infection. Nat Med.

[17] Sivro A (2018). Integrin α_4_β_7_ expression on peripheral blood CD4^+^ T cells predicts HIV acquisition and disease progression outcomes. Sci Transl Med.

[18] Frank I (2021). Blocking α_4_β_7_ integrin delays viral rebound in SHIV_SF162P3_-infected macaques treated with anti-HIV broadly neutralizing antibodies. Sci Transl Med.

[19] Di Mascio M (2019). Evaluation of an antibody to α_4_β_7_ in the control of SIVmac239-*nef-stop* infection. Science.

[20] Abbink P (2019). Lack of therapeutic efficacy of an antibody to α_4_β_7_ in SIVmac251-infected rhesus macaques. Science.

[21] Iwamoto N (2019). Blocking α_4_β_7_ integrin binding to SIV does not improve virologic control. Science.

[22] Johnson SD (2022). Early treatment with anti-α4β7 antibody facilitates increased gut macrophage maturity in SIV-infected rhesus macaques. Front Immunol.

[23] Wells CR (2021). Mechanistic basis of post-treatment control of SIV after anti-α4β7 antibody therapy. PLoS Comput Biol.

[24] Pino M (2020). Safety and immunological evaluation of Interleukin-21 Plus Anti-α4β7 mAb combination therapy in Rhesus Macaques. Front Immunol.

[25] Szabo SDJ (2000). A novel transcription factor, T-bet, directs Th1 lineage commitment. Cell.

[26] Hersperger AR (2011). Increased HIV-specific CD8^+^ T-cell cytotoxic potential in HIV elite controllers is associated with T-bet expression. Blood.

[27] Strengell M (2002). IL-21 up-regulates the expression of genes associated with innate immunity and Th1 response. J Immunol.

[28] Porichis F, Kaufmann DE (2012). Role of PD-1 in HIV pathogenesis and as target for therapy. Curr HIV/AIDS Rep.

[29] Kao C (2011). Transcription factor T-bet represses expression of the inhibitory receptor PD-1 and sustains virus-specific CD8^+^ T cell responses during chronic infection. Nat Immunol.

[30] Ananthakrishnan AN (2017). Gut microbiome function predicts response to anti-integrin biologic therapy in inflammatory bowel diseases. Cell Host Microbe.

[31] Siddiqui S (2020). Alterations of the gut bacterial microbiota in rhesus macaques with SIV infection and on short- or long-term antiretroviral therapy. Sci Rep.

[32] Vujkovic-Cvijin I, Somsouk M (2019). HIV and the gut microbiota: composition, consequences, and avenues for amelioration. Curr HIV/AIDS Rep.

[33] Andoh A, Nishida A (2023). Alteration of the gut microbiome in inflammatory bowel disease. Digestion.

[34] Nishida A (2018). Gut microbiota in the pathogenesis of inflammatory bowel disease. Clin J Gastroenterol.

[35] Stojanov S (2020). The influence ofprobiotics on the firmicutes/bacteroidetes ratio in the treatment of obesity and inflammatory bowel disease. Microorganisms.

[36] Mehraj V (2016). The plasma levels of soluble ST2 as a marker of gut mucosal damage in early HIV infection. AIDS.

[37] Brenchley JM (2006). Microbial translocation is a cause of systemic immune activation in chronic HIV infection. Nat Med.

[38] Dillon SM (2017). Low abundance of colonic butyrate-producing bacteria in HIV infection is associated with microbial translocation and immune activation. AIDS.

[39] Montalban-Arques A (2021). Commensal Clostridiales strains mediate effective anti-cancer immune response against solid tumors. Cell Host Microbe.

[40] Chomont N (2009). HIV reservoir size and persistence are driven by T cell survival and homeostatic proliferation. Nat Med.

[41] Reeves DB (2023). Estimating the contribution of CD4 T cell subset proliferation and differentiation to HIV persistence. Nat Commun.

[42] Muthumani K (2008). Human immunodeficiency virus type 1 Nef induces programmed death 1 expression through a p38 mitogen-activated protein kinase-dependent mechanism. J Virol.

[43] Jimenez-Leon MR (2024). Vedolizumab and ART in recent HIV-1 infection unveil the role of α4β7 in reservoir size. JCI Insight.

[44] Paiardini M (2011). Low levels of SIV infection in sooty mangabey central memory CD4^+^ T cells are associated with limited CCR5 expression. Nat Med.

[45] Brenchley JM (2012). Differential infection patterns of CD4^+^ T cells and lymphoid tissue viral burden distinguish progressive and nonprogressive lentiviral infections. Blood.

[46] Estes JD (2008). Early resolution of acute immune activation and induction of PD-1 in SIV-infected sooty mangabeys distinguishes nonpathogenic from pathogenic infection in rhesus macaques. J Immunol.

[47] Huot N (2018). Lymph node cellular and viral dynamics in natural hosts and impact for HIV cure strategies. Front Immunol.

[48] Klatt NR (2014). Limited HIV infection of central memory and stem cell memory CD4^+^ T cells is associated with lack of progression in viremic individuals. PLoS Pathog.

[49] Sáez-Cirión A (2013). Post-treatment HIV-1 controllers with a long-term virological remission after the interruption of early initiated antiretroviral therapy ANRS VISCONTI Study. PLoS Pathog.

[50] Descours B (2012). Immune responses driven by protective human leukocyte antigen alleles from long-term nonprogressors are associated with low HIV reservoir in central memory CD4 T cells. Clin Infect Dis.

[51] Hatano H (2013). Cell-based measures of viral persistence are associated with immune activation and programmed cell death protein 1 (PD-1)-expressing CD4^+^ T cells. J Infect Dis.

[52] Nie K (2021). *Roseburia intestinalis*: a beneficial gut organism from the discoveries in genus and species. Front Cell Infect Microbiol.

[53] Ortiz AM (2022). Butyrate administration is not sufficient to improve immune reconstitution in antiretroviral-treated SIV-infected macaques. Sci Rep.

[54] Comalada M (2006). The effects of short-chain fatty acids on colon epithelial proliferation and survival depend on the cellular phenotype. J Cancer Res Clin Oncol.

[55] Mariadason JM (2001). Resistance to butyrate-induced cell differentiation and apoptosis during spontaneous Caco-2 cell differentiation. Gastroenterology.

[56] Coutzac C (2020). Systemic short chain fatty acids limit antitumor effect of CTLA-4 blockade in hosts with cancer. Nat Commun.

[57] Johnson SD (2024). Dual role for microbial short-chain fatty acids in modifying SIV disease trajectory following anti-α4β7 antibody administration. Ann Med.

[58] Clasen SDJ (2023). Silent recognition of flagellins from human gut commensal bacteria by Toll-like receptor 5. Sci Immunol.

[59] Quan Y (2018). Roseburia intestinalis-derived flagellin is a negative regulator of intestinal inflammation. Biochem Biophys Res Commun.

[60] Ruan G (2022). *Roseburia intestinalis* and its metabolite butyrate inhibit colitis and upregulate TLR5 through the SP3 signaling pathway. Nutrients.

[61] Vanhamel J (2019). Establishment of latent HIV-1 reservoirs: what do we really know?. J Virus Erad.

[62] Patterson AM (2017). Human gut symbiont *Roseburia*
*hominis* promotes and regulates innate immunity. Front Immunol.

[63] Harper J (2022). Interleukin-10 contributes to reservoir establishment and persistence in SIV-infected macaques treated with antiretroviral therapy. J Clin Invest.

[64] McGary CS (2017). CTLA-4^+^PD-1^–^ Memory CD4^+^ T cells critically contribute to viral persistence in antiretroviral therapy-suppressed, SIV-infected Rhesus Macaques. Immunity.

[65] Paramsothy S (2019). Specific bacteria and metabolites associated with response to Fecal Microbiota Transplantation in patients with ulcerative colitis. Gastroenterology.

[66] Serrano-Villar S (2021). Fecal microbiota transplantation in HIV: A pilot placebo-controlled study. Nat Commun.

[67] Vujkovic-Cvijin I (2017). Limited engraftment of donor microbiome via one-time fecal microbial transplantation in treated HIV-infected individuals. Gut Microbes.

[68] Hensley-McBain T (2016). Effects of Fecal Microbial Transplantation on microbiome and immunity in Simian Immunodeficiency Virus-Infected macaques. J Virol.

[69] Isnard S (2022). Camu Camu effects on microbial translocation and systemic immune activation in ART-treated people living with HIV: protocol of the single-arm non-randomised Camu Camu prebiotic pilot study (CIHR/CTN PT032). BMJ Open.

[70] Messaoudene M (2022). A natural polyphenol exerts antitumor activity and circumvents Anti-PD-1 resistance through effects on the gut microbiota. Cancer Discov.

[71] Rasmussen TA (2021). Impact of Anti-PD-1 and Anti-CTLA-4 on the Human Immunodeficiency Virus (HIV) reservoir in people living with HIV with cancer on antiretroviral therapy: the AIDS Malignancy Consortium 095 Study. Clin Infect Dis.

[72] Dyavar Shetty R (2012). PD-1 blockade during chronic SIV infection reduces hyperimmune activation and microbial translocation in rhesus macaques. J Clin Invest.

[73] Velu V (2009). Enhancing SIV-specific immunity in vivo by PD-1 blockade. Nature.

[74] Velu V (2022). PD-1 blockade following ART interruption enhances control of pathogenic SIV in rhesus macaques. Proc Natl Acad Sci U S A.

[75] Lavolé A (2018). PD-1 blockade in HIV-infected patients with lung cancer: a new challenge or already a strategy?. Ann Oncol.

[76] Gay CL (2017). Clinical trial of the Anti-PD-L1 antibody BMS-936559 in HIV-1 infected participants on suppressive antiretroviral therapy. J Infect Dis.

[77] Abu-Sbeih H (2018). Outcomes of vedolizumab therapy in patients with immune checkpoint inhibitor-induced colitis: a multi-center study. J Immunother Cancer.

[78] Fidelle M (2023). A microbiota-modulated checkpoint directs immunosuppressive intestinal T cells into cancers. Science.

[79] Cecchinato V (2008). Immune activation driven by CTLA-4 blockade augments viral replication at mucosal sites in simian immunodeficiency virus infection. J Immunol.

[80] Hryniewicz A (2006). CTLA-4 blockade decreases TGF-beta, IDO, and viral RNA expression in tissues of SIVmac251-infected macaques. Blood.

[81] Harper J (2020). CTLA-4 and PD-1 dual blockade induces SIV reactivation without control of rebound after antiretroviral therapy interruption. Nat Med.

[82] Colston E (2018). An open-label, multiple ascending dose study of the anti-CTLA-4 antibody ipilimumab in viremic HIV patients. PLoS One.

[83] Acharya A (2021). Chronic morphine administration differentially modulates viral reservoirs in a Simian Immunodeficiency Virus SIVmac251-infected Rhesus Macaque model. J Virol.

[84] Cline AN (2005). Highly sensitive SIV plasma viral load assay: practical considerations, realistic performance expectations, and application to reverse engineering of vaccines for AIDS. J Med Primatol.

[85] Li H (2016). Envelope residue 375 substitutions in simian-human immunodeficiency viruses enhance CD4 binding and replication in rhesus macaques. Proc Natl Acad Sci U S A.

[86] Dyavar SR (2018). Normalization of cell associated antiretroviral drug concentrations with a novel RPP30 droplet digital PCR assay. Sci Rep.

[87] Bender AM (2019). The landscape of persistent viral genomes in ART-treated SIV, SHIV, and HIV-2 infections. Cell Host Microbe.

