# Supplementary material for: IL-21 and anti-α4β7 dual therapy during ART promotes immunological and microbiome responses in SIV-infected macaques
Source: JCI Insight. 2025 Feb 4;10(6):e184491. doi: 10.1172/jci.insight.184491 (PMC11949015; doi:10.1172/jci.insight.184491)
Supplement: Supplemental data [file jciinsight-10-184491-s299.pdf]

Interleukin-21 and anti-α4β7 dual therapy during ART promotes immunological and microbiome responses in SIV-infected macaques

Samuel D. Johnson[1,7], Maria Pino[2,7], Arpan Acharya[1], Julien Clain[2], Deepanwita Bose[3], Kevin Nguyen[2], Justin Harper [2], Francois Villinger[3], Mirko Paiardini[2,4*], Siddappa N. Byrareddy[1,5,6,*]

1. Department of Pharmacology and Experimental Neuroscience, University of Nebraska Medical Center, Omaha, NE, United States

2. Division of Microbiology and Immunology, Emory National Primate Research Center, Emory University, Atlanta, GA, United States

3. New Iberia Research Center, University of Louisiana at Lafayette, New Iberia, Louisiana, USA.

4. Department of Pathology and Laboratory Medicine, Emory University School of Medicine, Atlanta, GA, United States

5. Department of Genetics, Cell Biology and Anatomy, University of Nebraska Medical Center, Omaha, NE, United States

6. Department of Biochemistry and Molecular Biology, University of Nebraska Medical Center, Omaha, NE, United States

7. Equally contributed

* For correspondence: Mirko Paiardini, mirko.paiardini@emory.edu and Siddappa N. Byrareddy, sid.byrareddy@unmc.edu

Supplemental Figures and Tables

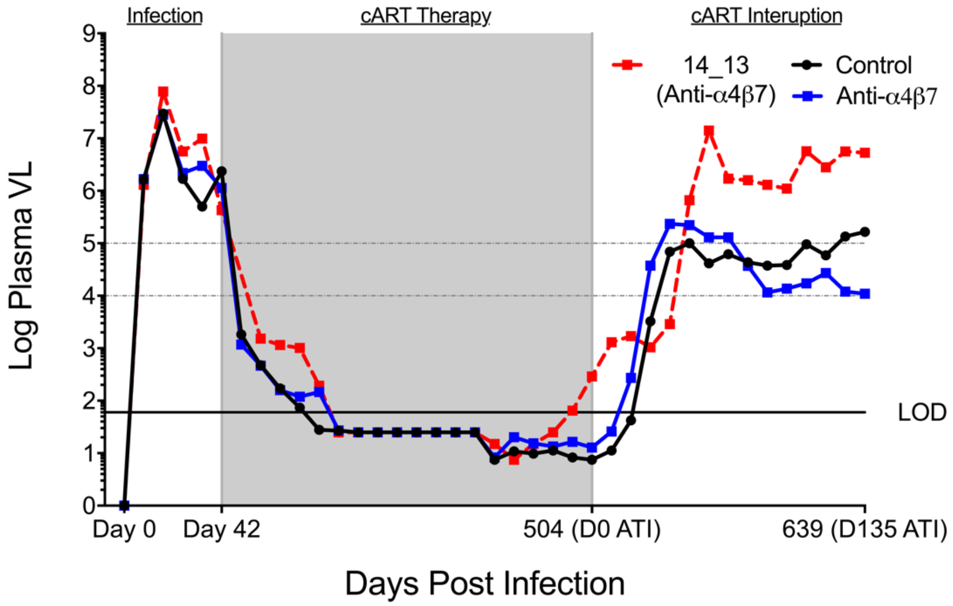

Supplemental Figure 1: Viral Loads with 14_13 shown separately from other treated individuals

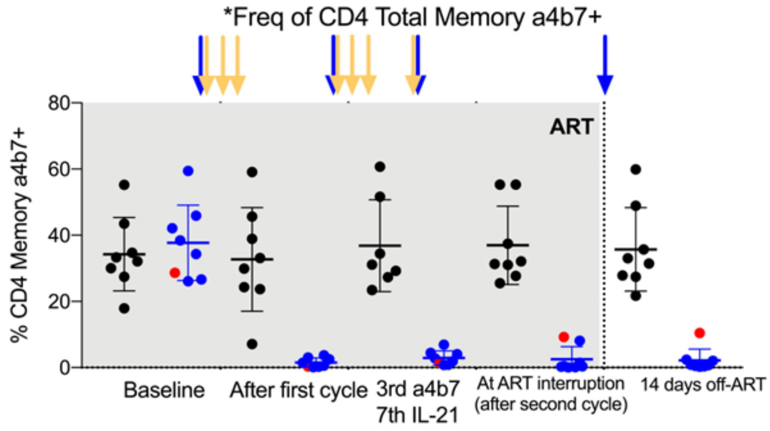

Supplemental Figure 2: Dual therapy effectively masks α4β7 integrin on CD4. Note: Excluded RM 14_13 is in red.

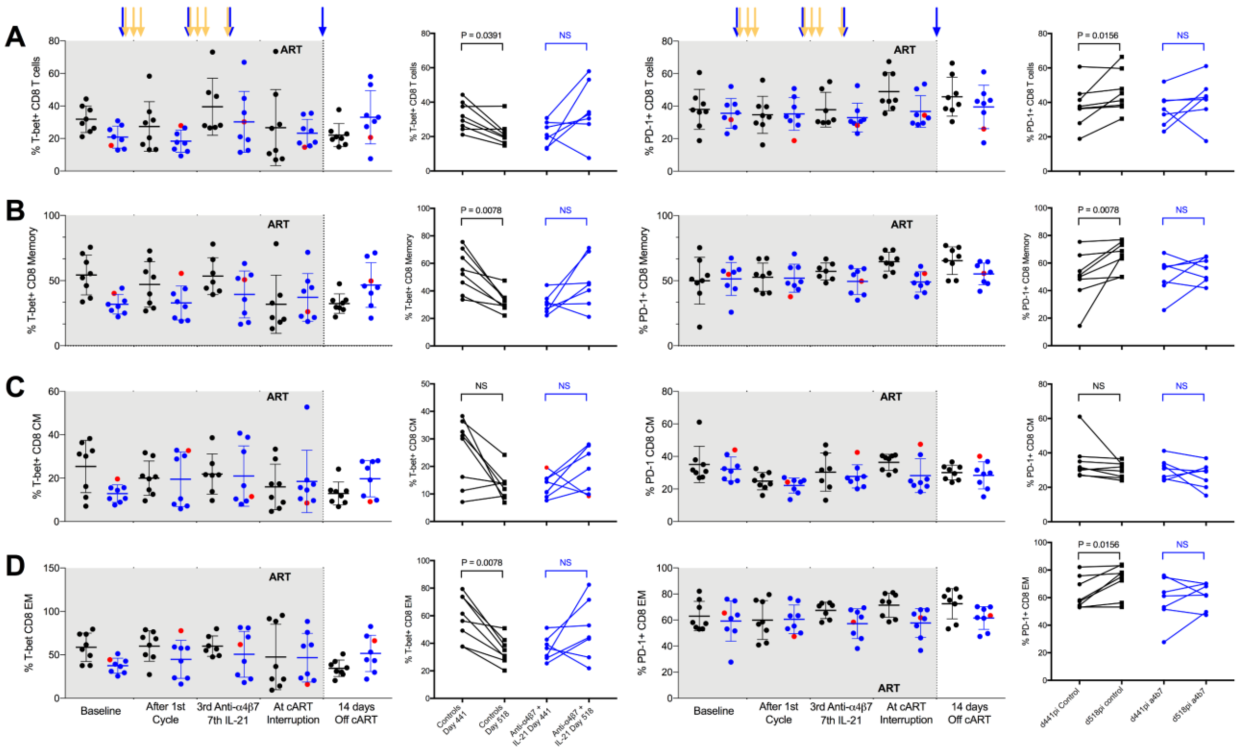

Supplemental Figure 3: T-bet and PD-1 expression on CD8+ T cell subsets in blood, including (A) total CD8+, (B) CD8+ memory, (C) CD8+ central memory, and (D) CD8+ effector memory T cells.

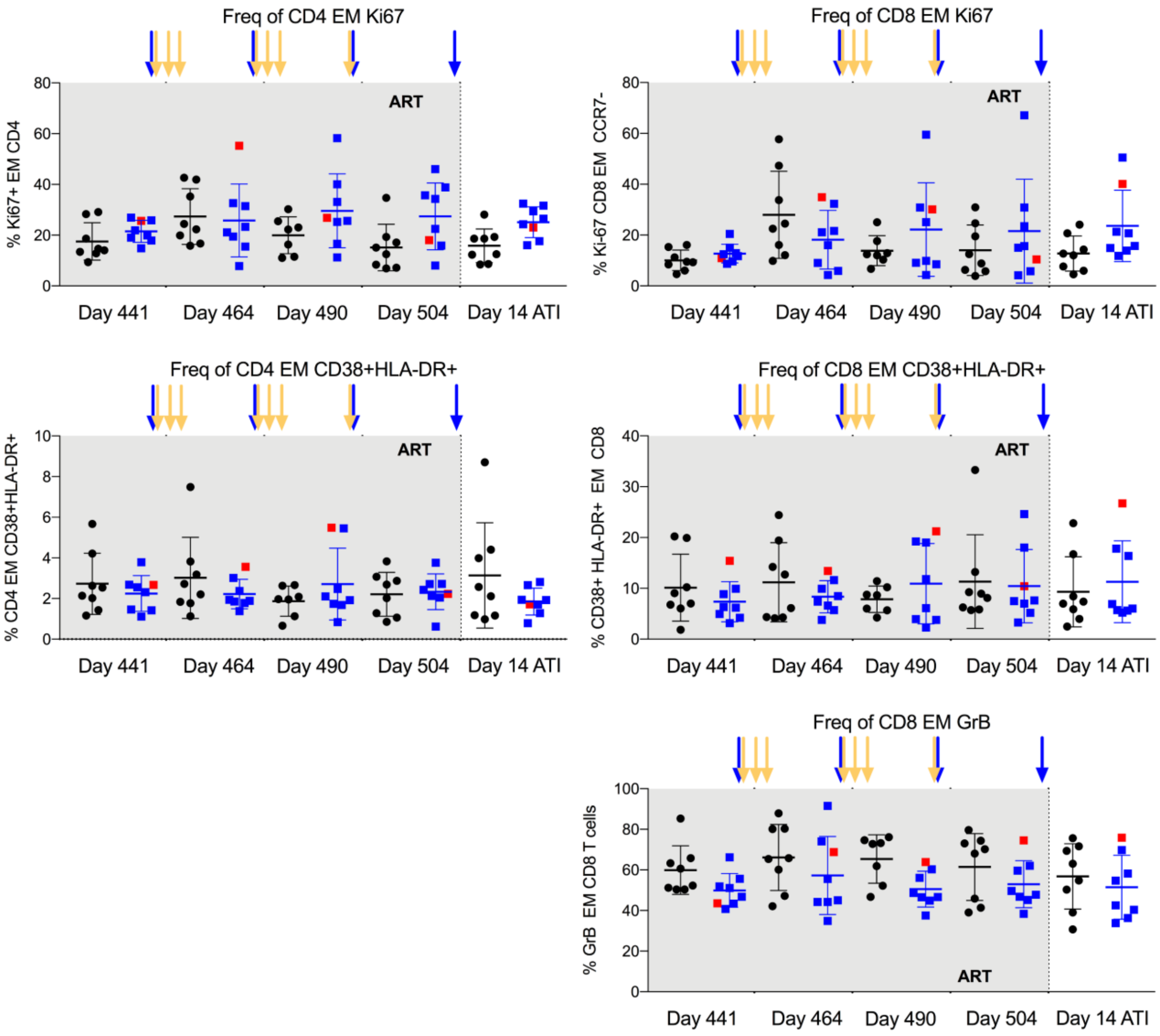

Supplemental Figure 4: Expression of Ki67, activation markers CD38 and HLA-DR, and Granzyme B on peripheral CD4+ and CD8+ effector memory T cell subsets.

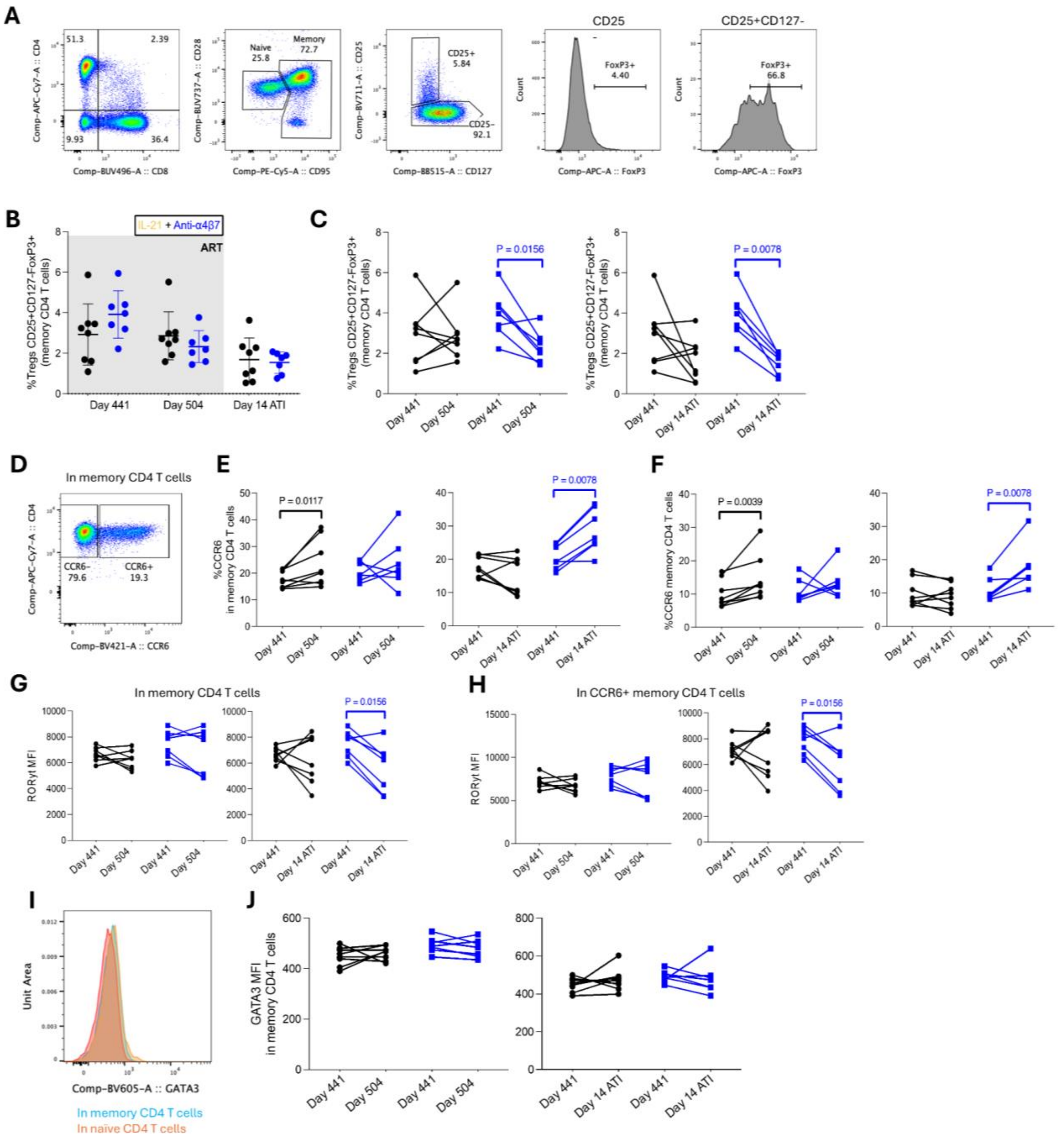

Supplemental Figure 5: Peripheral CD4+ T cell immunophenotyping. A) Gating strategy for T regulatory cells (CD25+CD127-FOXP3+); B, C) Longitudinal percent peripheral T regulatory T cells; D) Gating strategy for CCR6+ memory cells; E) percent CCR6+ of memory cells; F) percent CCR6+ memory cells of total peripheral CD4+ T cells; RORγt MFI on G) memory and H) CCR6+ memory CD4+ T cells; and I, J) GATA3 MFI on memory CD4+ T cells.

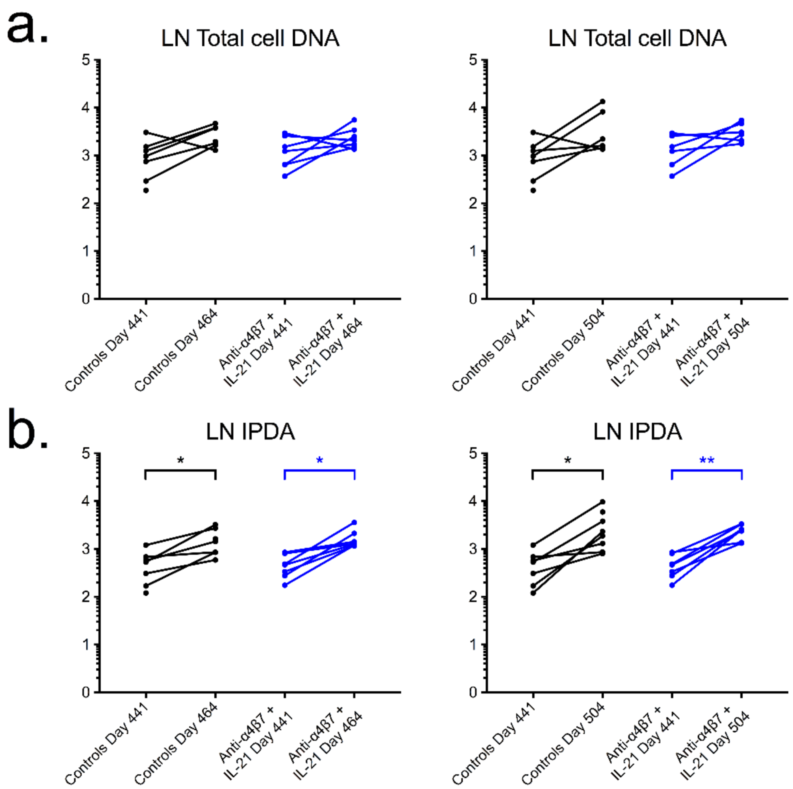

Supplemental Figure 6: Measures of lymph node reservoir size, including (A) total SIVgag DNA copies normalized and (B) intact proviral DNA Assay (IPDA), each normalized to 10^6 cells.

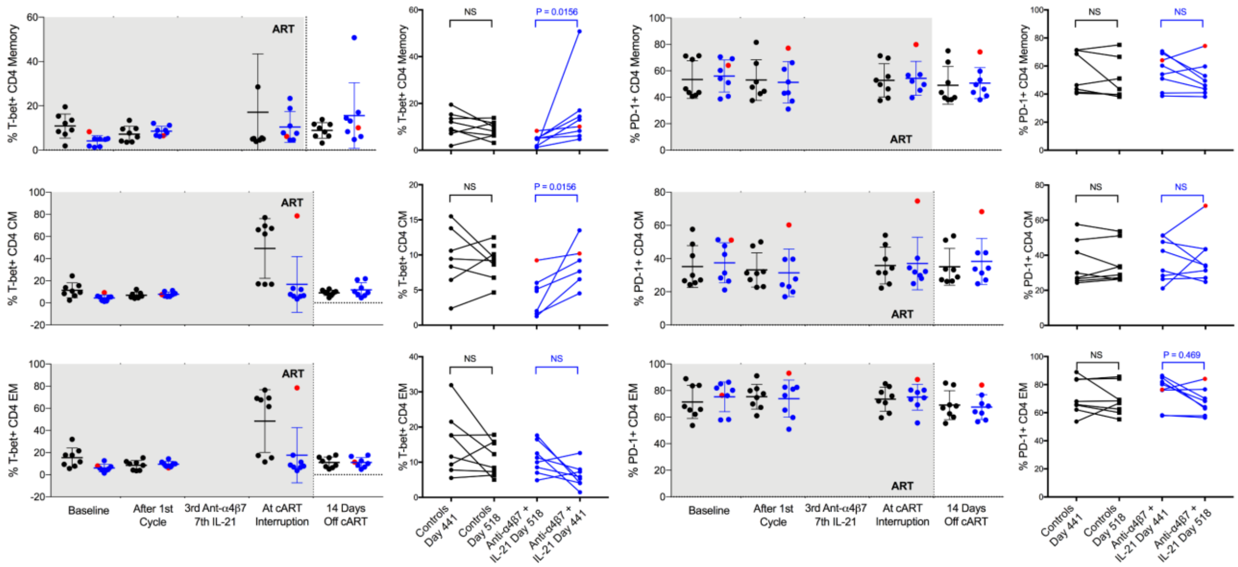

Supplemental Figure 7: LN Immune cell phenotypes, including T-bet and PD-1 expression. Statistics performed excluding 14_13 (red).

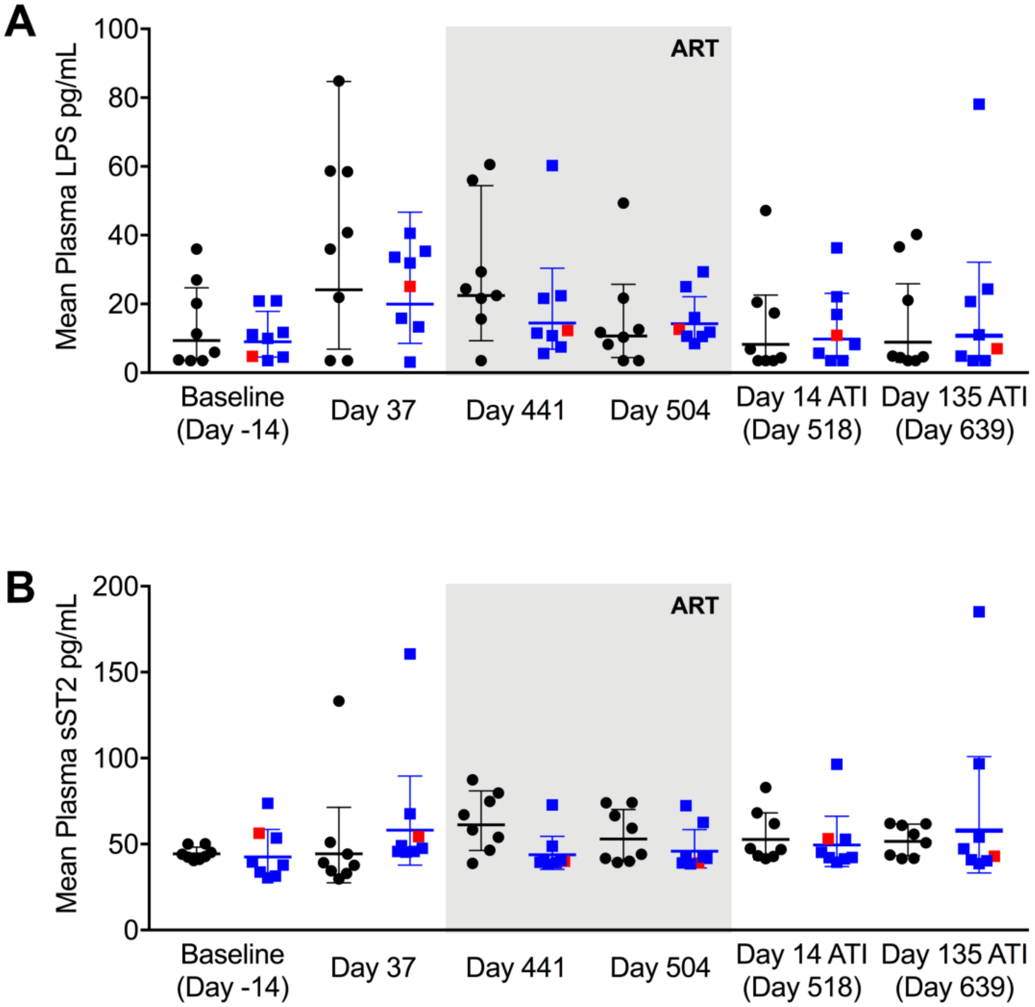

Supplemental Figure 8: Longitudinal plasma biomarkers of gut barrier function, including A) lipopolysaccharide and B) soluble ST2 levels

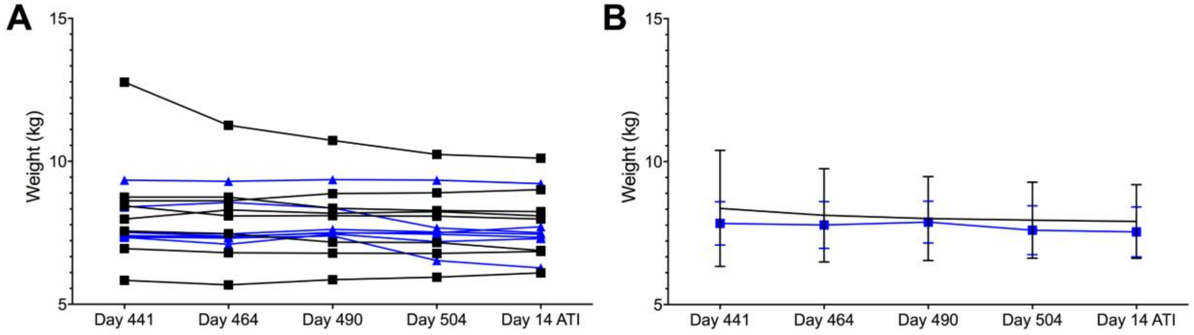

Supplemental Figure 9: Longitudinal macaque weights

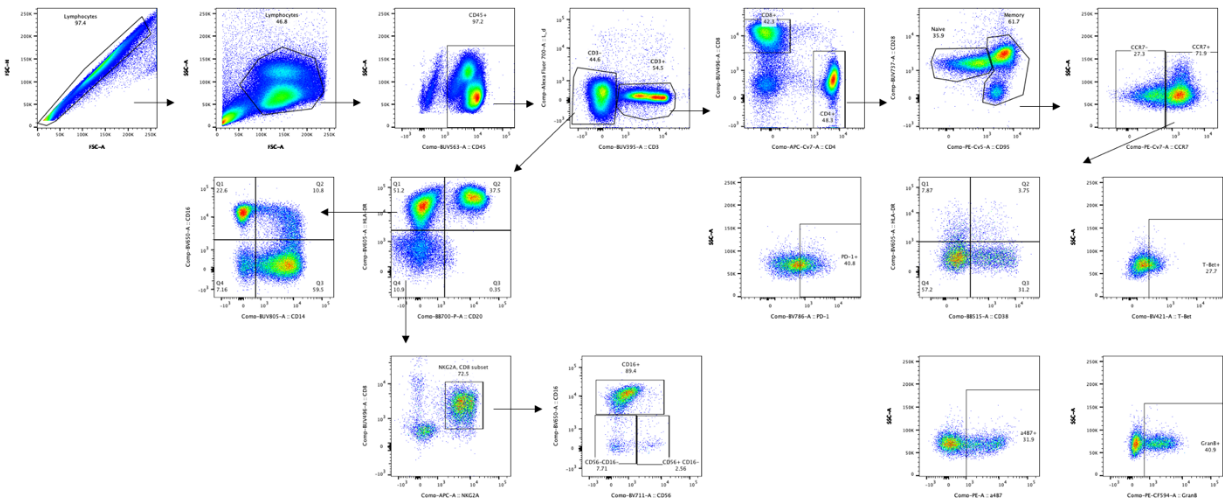

Supplemental Figure 10: Gating Strategy

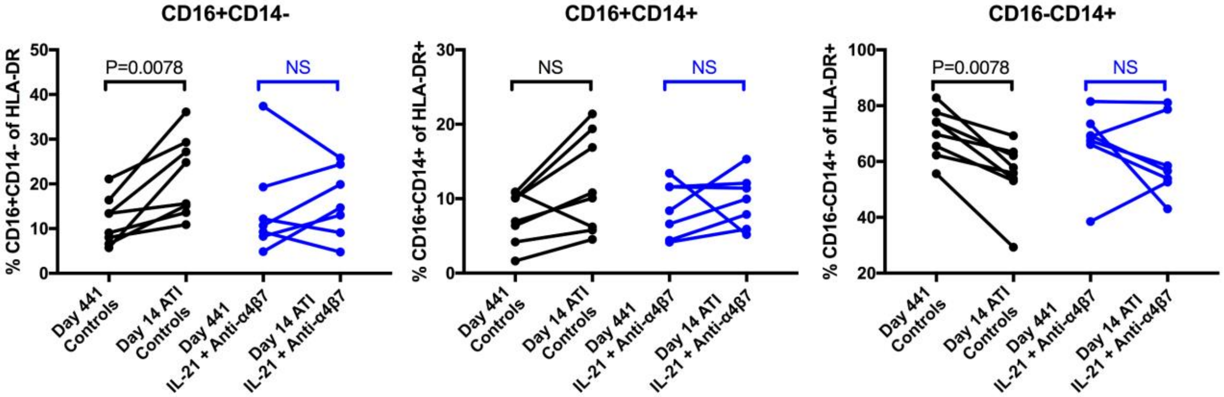

Supplemental Figure 11: Peripheral monocyte subsets

	Animal code	Age at infection (months)	Gender	Peak viremia VL (Log10 copies/mL)	Setpoint VL (d42pi; Log10 copies/mL)	freq CD4 CD95+ a4b7bright pre-infection	freq CD4 CD95+ a4b7bright last time	ADA mAb a4b7	A01
**Control group**	14C002	48	Female	8.23	6.45	21.1	19.8	**+**	-
	RFm12	117	Female	7.32	5.90	31.8	25.5	**+**	-
	13C081	57	Female	7.40	7.15	21.5	23.3	**+**	-
	RCr11	140	Female	6.98	6.32	29.8	29.6	-	+
	14C122	45	Female	7.18	5.83	35.6	25.6	-	-
	RIl13	104	Female	7.36	5.04	19.8	23.8	-	-
	14C289	44	Female	7.63	7.38	19.2	27.7	**+**	-
	14C141	46	Male	7.64	6.90	16.6	21.9	**+**	-
**a4b7+IL-21 group**	14_134	43	Female	6.70	5.28	23	20.6	-	-
	11_32	80	Female	7.56	6.18	37.7	41.5	-	-
	14C066	45	Male	7.46	7.04	22.1	27.2	-	-
	14_13	45	Female	7.89	5.63	24.1	24.6	-	-
	RFd12	128	Female	7.69	6.79	17.7	28.6	-	+
	RHy13	93	Female	7.88	6.11	25.2	24.5	-	-
	13M257	55	Female	7.71	6.83	22.3	20.2	-	-
	RWn12	118	Female	7.18	4.15	36.5	31.9	-	-

Supplemental Table 1: Rhesus macaque characteristics, including criteria utilized for appropriate stratification of animals into groups. “ADA mAb a4b7” indicates whether the RM tested +/- for pre-existent anti-anti-α4β7-specific antibodies, prior to infection. “A01” indicates MHC haplotype *Mamu*-A*01^{+}.

Phenotype panel 1					
**Vendor**	**Antibody**	**Fluorochrome**	**Cat #**	**Clone**	**Isotype**
Stemcell	CD38-FITC	FITC	60131FI	AT1	Mouse IgG1, κ
Biolegend	CD20-PerCpCy5.5	PerCpCy5.5	302326	2H7	Mouse IgG2b, κ
Beckman Coulter	NKG2a-APC	APC	A60797	Z199	IgG2b Mouse
Biolegend	CD4-APCCy7	APC Cy7	317418	OKT4	Mouse IgG2b, κ
NHP Reagent Resource	alpha4/beta 7 -PE	PE	PR-1421	A4B7R1	Rhesus IgG1 Fc
BD	CD45	BUV563	741414	D058-1283	Mouse IgG1, κ
BD	CD28-BUV737	BUV737	564438	CD28.2	Mouse C3H x BALB/c IgG1, κ
BD	CCR7-Cy7PE	Cy7PE	557648	3D12	Rat anti-human
BD	CD14-BUV805	BUV805	612902	M5E2	Mouse IgG2a, κ
Biolegend	T-bet	BV421	644816	4B10	Mouse IgG1, κ
BD	KI67-BV480	BV480	566109	B56	Mouse IgG1, κ
BD	HLADR-BV605	BV605	562845	G46-6	Mouse IgG2a, κ
BD	CD16-BV650	BV650	563692	3G8	Mouse BALB/c x DBA/2, also known as CD2F1 or CDF1 IgG1, κ
Biolegend	PD1-BV785	BV785	329930	EH12.2H7	Mouse IgG1, κ
BD	CD3-BUV395	BUV395	564117	SP34-2	Mouse BALB/c IgG1, λ
BD	CD8-BUV496	BUV496	564804	RPA-T8	Mouse IgG1, κ
BD	CD56-BV711	BV711	740781	B159	Mouse IgG1, κ
eBioscience	CXCR5-PE	PE	12-9185-42	MU5UBEE	Mouse / IgG2b, kappa
Invitrogen	GranB-PETR	PETR	GRB17	GB11	Mouse / IgG1
BD	CD95-Cy5PE	CY5PE	559773	DX2	Mouse C3H, also known as C3H/He, C3H/Bi IgG_1, κ

Supplemental Table 2: Antibody list used for fresh PBMCs and LN cells

Phenotype Panel 2

Vendor	Antibody	Fluorochrome	Cat #	Clone	Isotype
Thermofisher Scientific	CD127	Alexa Fluor 488	53-1278-42	eBioRDR5	Mouse IgG1, κ
BD	CCR6	BV421	565925	11A9	Mouse IgG1, κ
Thermofisher Scientific	GATA3	BV605	406-9966-42	TWAJ	Rat IgG2b, k
Biolegend	CD25	BV711	302636	BC96	Mouse IgG1, κ
Thermofisher Scientific	RORyt	PE	12-6988-82	AFKJS-9	Rat IgG2b, k
Biolegend	CD95	PE-Cy5	305610	DX2	Mouse IgG1, κ
BD	CD3	BUV395	564117	SP34-2	Mouse BALB/c IgG1, λ
BD	CD8	BUV496	612942	RPA-T8	Mouse IgG1, κ
BD	CD28	BUV737	612815	CD28.2	Mouse C3H x BALB/c IgG1, κ
Biolegend	FoxP3	Alexa Fluor® 647	320014	150D	Mouse IgG1, κ
Biolegend	CD4	APC-Cy7	317418	OKT4	Mouse IgG2b, κ
BD		Fixable Viability Stain 700	564997		

Supplemental Table 3: Second antibody list used for frozen PBMCs
